# 2-(3-Bromophenyl)-8-fluoroquinazoline-4-carboxylic Acid as a Novel and Selective Aurora A Kinase Inhibitory Lead with Apoptosis Properties: Design, Synthesis, In Vitro and In Silico Biological Evaluation

**DOI:** 10.3390/life12060876

**Published:** 2022-06-10

**Authors:** Mohamed H. Elsherbeny, Usama M. Ammar, Magda H. Abdellattif, Mohammed A. S. Abourehab, Ahmed Abdeen, Samah F. Ibrahim, Doaa Abdelrahaman, Wessam Mady, Eun Joo Roh, Ahmed Elkamhawy

**Affiliations:** 1 Chemical and Biological Integrative Research Center, Korea Institute of Science and Technology (KIST), Seoul 02792, Korea; mohamed.alsherbeny@pharma.asu.edu.eg; 2 Division of Bio-Medical Science & Technology, KIST School, University of Science and Technology, Seoul 02792, Korea; 3 Pharmaceutical Chemistry Department, Faculty of Pharmacy, Ahram Canadian University, Giza 12566, Egypt; 4 Strathclyde Institute of Pharmacy and Biomedical Sciences, University of Strathclyde, 161 Cathedral Street, Glasgow G4 0NR, UK; usama.ammar@strath.ac.uk; 5 Department of Chemistry, College of Science, Taif University, P.O. Box 11099, Taif 21944, Saudi Arabia; m.hasan@tu.edu.sa; 6 Department of Pharmaceutics, College of Pharmacy, Umm Al-Qura University, Makkah 21955, Saudi Arabia; maabourehab@uqu.edu.sa; 7 Department of Pharmaceutics and Industrial Pharmacy, College of Pharmacy, Minia University, Minia 61519, Egypt; 8 Department of Forensic Medicine and Toxicology, Faculty of Veterinary Medicine, Benha University, Toukh 13736, Egypt; ahmed.abdeen@fvtm.bu.edu.eg; 9 Department of Clinical Sciences, College of Medicine, Princess Nourah bint Abdulrahman University, P.O. Box 84428, Riyadh 11671, Saudi Arabia; sfibrahim@pnu.edu.sa (S.F.I.); dsabdelrahman@pnu.edu.sa (D.A.); 10 Center of Excellence in Genomic Medicine Research, King Abdulaziz University, Jeddah 21589, Saudi Arabia; wmadi@kau.edu.sa; 11 BK21 FOUR Team and Integrated Research Institute for Drug Development, College of Pharmacy, Dongguk University-Seoul, Goyang 10326, Korea; a_elkamhawy@mans.edu.eg; 12 Department of Pharmaceutical Organic Chemistry, Faculty of Pharmacy, Mansoura University, Mansoura 35516, Egypt

**Keywords:** quinazoline, Aurora A, kinases, anticancer, molecular docking, cell cycle, apoptosis

## Abstract

New quinazoline derivatives were designed based on the structural modification of the reported inhibitors to enhance their selectivity toward Aurora A. The synthesized compounds were tested over Aurora A, and a cytotoxicity assay was performed over NCI cell lines to select the best candidate for further evaluation. Compound **6e** (2-(3-bromophenyl)-8-fluoroquinazoline-4-carboxylic acid) was the most potent compound among the tested derivatives. A Kinase panel assay was conducted for compound **6e** over 14 kinases to evaluate its selectivity profile. Further cell cycle and apoptosis analysis were evaluated for compound **6e** over the MCF-7 cell line at its IC_50_ of 168.78 µM. It arrested the cell cycle at the G1 phase and induced apoptosis. Molecular docking was performed to explore the possible binding mode of compound **6e** into the active site. It showed significant binding into the main pocket in addition to potential binding interactions with the key amino acid residues. Accordingly, compound **6e** can be considered a potential lead for further structural and molecular optimization of the quinazoline-based carboxylic acid scaffold for Aurora A kinase selective inhibition with apoptosis properties.

## 1. Introduction

**Figure 1:**
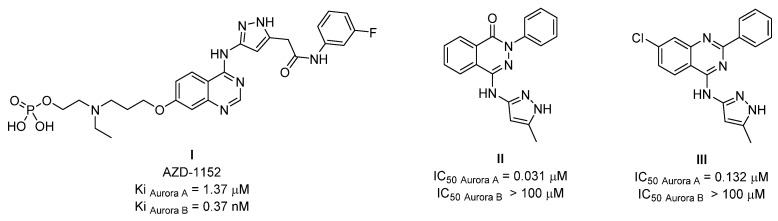
Reported Aurora A inhibitors.

Cancer is considered the second most common cause of death worldwide and is characterized by uncontrolled cell proliferation [1,2]. Several kinases have been found to have a key role in cell growth and proliferation [3]. Aurora kinases (Aurora A, B and C) play a significant role in cell cycle regulation and mitotic spindle development. Aurora kinase is a class of serine/threonine kinase that regulates cell division. These kinases are regulatory proteins involved in centrosome maturation, chromosome alignment, chromosome segregation and cytokinesis [4]. Aurora kinase dysfunction may lead to critical, unstable chromosome content and may lead to cancer disease [5,6]. Aurora A kinase is a member of the Aurora kinase family that is extensively overexpressed in human malignancies [7]. As a result, Aurora A kinase has been proposed as a promising anticancer target for human cancer treatment. Inhibition of protein kinases via the ATP-binding site (adenosine triphosphate) has emerged as a promising route for treating cancer [1]. Aurora kinases have sparked a lot of interest as anticancer targets throughout recent decades [8,9,10]. The discovery of a large number of small-molecule inhibitors of Aurora kinases, the majority of which inhibit both isoforms A and B, led to the discovery of Aurora kinase modulators as cancer-treating agents. One example of a non-selective Aurora A and B inhibitor is a quinazoline-based derivative (AZD-1152, **I**) with different binding motifs around the central core [11,12]. However, a recent study found that Aurora B suppression is linked to a number of side effects, including the production of unstable polyploid cells, which could stimulate carcinogenesis [13]. Therefore, the selective inhibition of Aurora A over Aurora B isoform has been considered an attractive anticancer target for the treatment of human cancers [7]. A number of Aurora A kinase inhibitors have been developed (phthalazine-based derivative, **II** [14] and quinazoline-based derivatives, **III** [3], which showed a selective profile towards Aurora A over Aurora B isoform (Figure 1).

It was noticed that the selective inhibitors (**II** and **III**) shared a similarly sized fused bicyclic core scaffold with two nitrogen hetero atoms (HBAs, hydrogen bond acceptors) with the non-selective inhibitor (AZD-1152, **I**). However, the selective agents (**II** and **III**) showed structural simplification around the central core compared to non-selective inhibitors (AZD-1152, **I**). It was suggested that the binding active site of Aurora A kinase has limited-sized access compared to that of Aurora B kinase. As a result, the small-sized derivatives may accommodate the Aurora A active site with appropriate binding forces. Therefore, our research group was interested in investigating the Aurora A inhibitory activity of small-sized substitution-containing quinazoline central scaffolds (**5b**, **6a**–**e** and **7a**,**b**).

**Figure 2:**
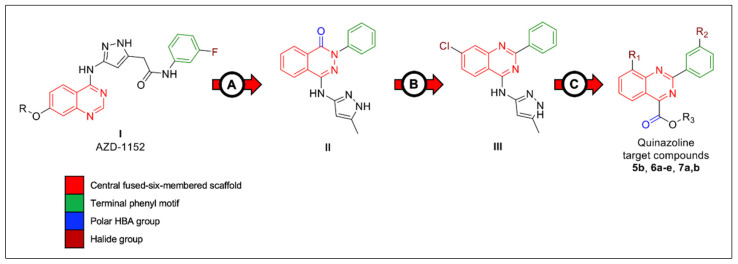
Rational design of synthesized compounds. (**A**) Structural simplification was applied to the quinazoline-based scaffold (**I**, AZD-1152) to enhance the selectivity profile towards Aurora A over Aurora B kinase enzyme. Both terminal long aliphatic chain and amide spacer were omitted. In addition, the central fused six-membered ring was replaced with its ring isostere (phthalazine ring). Terminal phenyl ring was directly attached to the central scaffold; (**B**) structural modification was designed to introduce the quinazoline ring as a core central scaffold. An additional halogen group (Cl) was introduced into the quinazoline ring; (**C**) the current derivatives (**5b**, **6a**–**e** and **7a**,**b**) were designed to keep the quinazoline ring as the main core scaffold. A small halogen group (F) was introduced to the quinazoline ring to explore any additional binding to the key amino acid residues in the active site of Aurora A kinase without any steric hindrance. Additional groups were introduced into the terminal phenyl ring (Me, Cl and Br). Terminal polar groups were introduced to be a potential binding motif for the solvent-exposure area (free carboxylic acid, **6a**–**e**; and ester-based substitution, **5b** and **7a**,**b**).

In addition, we designed a series of compounds with differing sized halogen atoms that can afford additional binding(s) to the key amino acids inside the active site of Aurora A kinase through halogen interaction. Furthermore, the terminal free carboxylic group was incorporated in the designed derivatives to be a potential solvent-exposed binding group (**6a**–**e**). Additionally, a group of ester-based derivatives (**5b** and **7a**,**b**) were designed in order to enhance the t-PSA (topological polar surface area) of these derivatives to investigate the effect of enhanced t-PSA on both the inhibitory activity as well as the cellular induction through the lipophilic membrane (Figure 2). In vitro screening (including kinase inhibitory assay, cytotoxic assay, cell cycle arrest analysis and apoptosis assay) and in silico molecular docking screening were conducted on the designed compounds to evaluate the inhibitory activity as well as the selectivity profile.

## 2. Results and Discussion

### 2.1. Chemistry

**Scheme 1:**
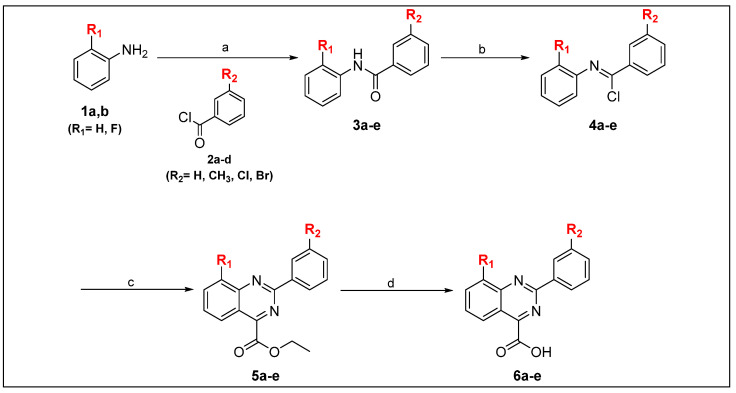
Reagents and conditions. (**a**) Et_3_N, DCM, 0 °C, 4 h; (**b**) SOCl_2_, reflux, 3 h; (**c**) EtOOC-CN, SnCl_4_, *o*-DCB, 140 °C, 15 min; (**d**) NaOH, EtOH, reflux, 2 h.

**Scheme 2:**
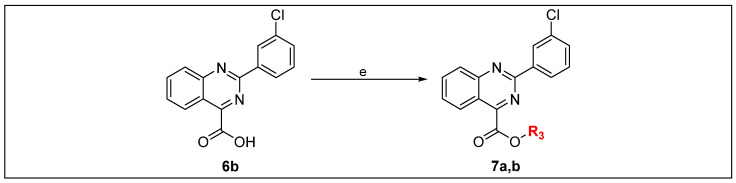
Reagents and conditions. (**e**) MeOH, (**7a**) *i*-PrOH, (**7b**) H_2_SO_4_, reflux, 18 h.

The final target compounds (**5b**, **6a**–**e** and **7a**,**b**) were synthesized as depicted in Scheme 1 and Scheme 2. Benzanilide derivatives (**3a**–**e**) were afforded through the Schotten–Baumann reaction [14]. Where appropriate, strong electrophilic acyl chlorides (**2a**,**b**) were coupled with selected primary amines (**1a**,**b**) in the presence of triethyl amine as a base. Compounds **3a**–**e** were refluxed with thionyl chloride to afford *N*-phenylbenzimidoyl chloride derivatives (**4a**–**e**). The cyclization of compounds **4a**–**e** was achieved by heating with ethyl cyanoformate in the presence of tin tetrachloride to afford compounds **5a**–**e**. Final target free carboxylic acid derivatives (**6a**–**e**) were afforded by basic hydrolysis of corresponding ethyl esters (**5a**–**e**). Ester-based derivatives (**7a**,**b**) were prepared by Fischer esterification of free carboxylic acid derivative (**6b**) with appropriate alcohols in the presence of a catalytic amount of sulfuric acid (Table 1).

### 2.2. In Vitro Screening

#### 2.2.1. Kinase Inhibitory Assay

An in vitro kinase inhibitory assay was conducted for the free carboxylic acid derivatives (**6a**–**e**) at single dose testing (10 µM) against Aurora A kinase enzyme (Table 2). The tested compounds showed different inhibitory activities. The results revealed that the compounds with halogen substitution at the terminal phenyl ring (**6b**,**e**) showed higher activities than that of unsubstituted (**6a**,**d**) and methyl-substituted derivative (**6c**). In addition, the fluorine atom in the main quinazoline scaffold possessed an additional inhibitory effect (compounds **6c**–**e**). Compound **6e** (bearing F atom in the core quinazoline scaffold and Br group at the terminal phenyl ring) exhibited the most potent inhibitory activity among the tested compounds (**6a**–**e**). The results showed that the structural modification that was applied to the reported Aurora A kinase inhibitors (**II** and **II**) to afford the current derivatives in this study will retain the inhibitory activity towards Aurora A kinase. The replacement of the terminal pyrazole ring with a free carboxylic group, along with the additional substitution with halogen groups (**6e**), maintained the ATP competitive binding into the active site of the target kinase (Aurora A). It was suggested that the terminal carboxylic group would provide the same HBD/HBA environment similar to the pyrazole ring, which may result in the same binding interactions with the key amino acid residues in the active site. However, the binding pattern may be altered (which will be discussed in the molecular docking section).

Compound **5b** (the ethyl ester precursor of the final free-carboxylic acid derivative **6b**) as well as compounds **7a**,**b** were designed, synthesized and tested to investigate the impact of the ester form of the target compounds on the inhibitory activity (Table 3). The results showed that the terminal free carboxylic group is essential for activity as the esterification of this group dropped the activity by five-fold. Compared to the free carboxylic-based derivatives, the masking effect of the free carboxylic group had a deteriorating effect on kinase inhibition (especially compound **7a,** which showed negative results). This revealed the crucial role of the free carboxylic group in the ATP competitive binding into the active site. It was suggested that the free carboxylic group share with essential HBD in the interaction with one of the key amino acid residues in the active site. Along with the essential HBD, the additional ethyl (**5b**), methyl (**7a**) and isopropyl (**7b**) groups may contribute to an unfavorable steric effect inside the active site with the core amino acid residue(s).

The most active compound (**6e**) among the tested compounds was selected for further investigation in an in vitro kinase panel assay to explore the selectivity profile of the Aurora A kinase enzyme. Fourteen kinase-related enzymes were selected to conduct the kinase panel assay at single dose testing (10 µM) (Table 4). The results revealed that compound **6e** showed poor inhibitory activities against the selected kinases (Activity% > 83%) in contrast to the inhibitory activity against Aurora A kinase enzyme (Activity% = 48.22%).

#### 2.2.2. Cytotoxicity Assay

In vitro cytotoxic screening of target compounds (free carboxylic acid derivatives, **6a**–**e**; and ester-based derivatives, **5b** and **7a**,**b**) was performed against NCI (National Cancer Institute) human cancer cell lines using a single dose assay (10 µM) (Table 5 and Tables S1–S8). The results showed that the tested compounds exhibited different cytotoxic activities. Moreover, the human CNS (central nervous system) cancer cell line (SNB-75) was the most sensitive cell line among the tested cell lines. Compound **6e** (the most active compound in the in vitro Aurora A kinase inhibitory assay) showed the most potent cytotoxic activities among the tested compounds. In line with their Aurora A kinase inhibitory results, the ester-based compounds (**5b** and **7a**,**b**) did not show any significant cytotoxic effect in the current study. It was proposed that the high t-PSA of ester-based derivatives failed to improve the cell induction and hence the cytotoxic activity.

#### 2.2.3. Cell Cycle Assay

**Figure 3:**
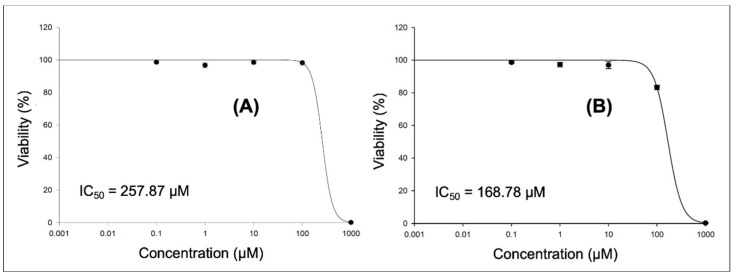
In vitro cytotoxic assay at 5-point assay of compound **6e** over human urinary bladder cancer cell line (T-24, (**A**)) and human breast cancer cell line (MCF-7, (**B**)).

In order to explore the effect of compound **6e** on the cell cycle, an initial in vitro cytotoxic assay was conducted over two available human cancer cell lines (urinary bladder cancer, T-24; and breast cancer, MCF-7) at five-point concentrations (0.1, 1, 10, 100 and 1000 µM) to calculate the IC_50_ (half-maximal inhibitory concentration) to be used in the cell cycle assay (Figure 3). Compound **6e** showed IC_50_ values of 257.87 and 168.78 µM over T-24 and MCF-7 cell lines, respectively.

**Figure 4:**
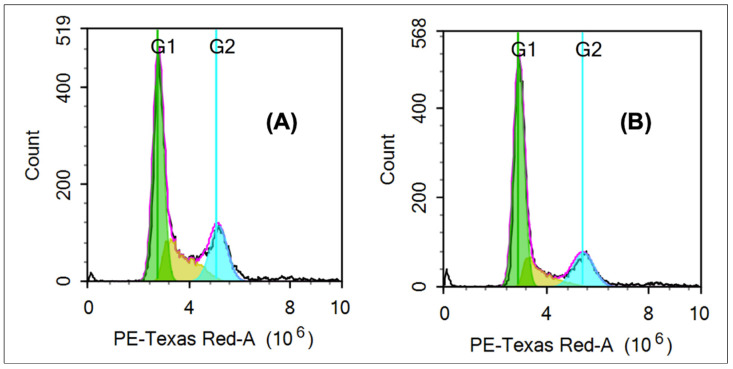
In vitro cell cycle cytotoxic assay of compound **6e** over human cancer cell line (MCF-7). Compound **6e** induced the cell cycle arrest at the G1 phase (**B**) using paclitaxel as a positive control (**A**). PE, phycoerythrin.

The cell cycle cytotoxicity assay was performed by treating the most sensitive cell line (breast cancer cell line, MCF-7) with compound **6e** at its IC_50_ for 24 h using paclitaxel (1 µM) as a positive control (Table 6). Cell cycle distribution was measured using flow cytometric analysis after staining the DNA (deoxyribonucleic acid) content of the treated cells with propidium iodide (Figure 4). Compound **6e** induced the increase in the cells of the G1 phase from 51.45% in control cells to 60.68% in the treated cells. Meanwhile, the cells of both S and G2 phases decreased to 17.47 and 18.29%, respectively, compared to that of control cells (22.27 and 21.34%, respectively). The results indicated that the cell cycle of MCF-7 cells was significantly arrested by compound **6e** at the DNA replication step rather than mitosis.

#### 2.2.4. Apoptosis

**Figure 5:**
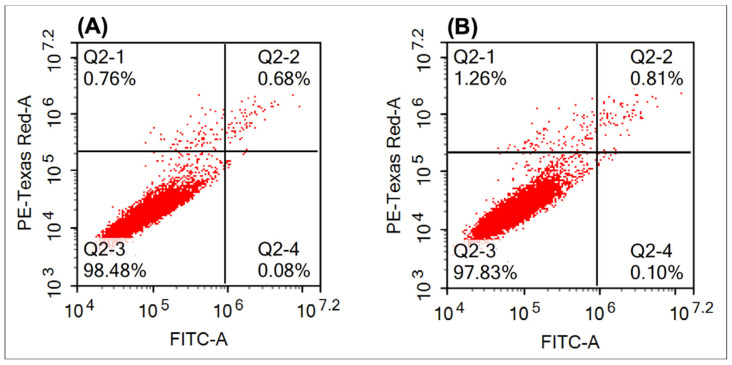
Effect of compound **6e** (**B**) on cellular apoptosis after 24 h co-culture with MCF-7 cell lines using doxorubicin as a positive control (**A**). FITC, fluorescein isothiocyanate.

The effect of compound **6e** on cellular apoptosis in a human breast cancer cell line (MCF-7) was evaluated at its IC_50_ using doxorubicin as a positive control (10 µM) after 24 h using flow cytometric analysis (Table 7). The results revealed that compound **6e** induced better cellular apoptosis (2.16%) compared to that of positive control (1.52%) after 24 h co-culture (Figure 5).

### 2.3. In Silico Screening

**Figure 6:**
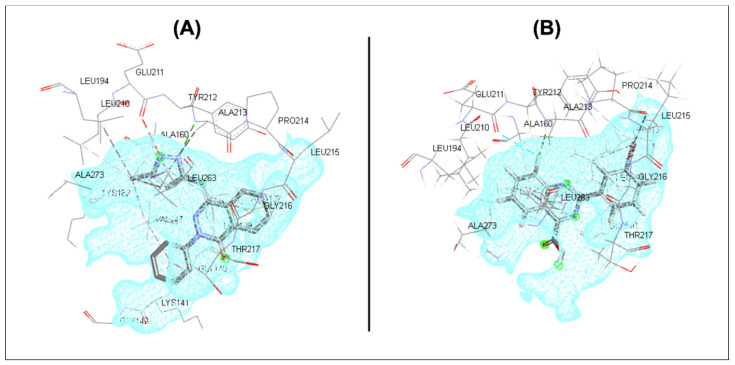
Three-dimensional binding mode into Aurora A active site (PDB ID: 3P9J). (**A**) reference ligand (**II**); (**B**) compound **6e**.

A molecular docking experiment was conducted for the tested compounds (**5b**, **6a**–**e** and **7a**,**b**) into the active site of the kinase domain in Aurora A kinase enzyme in order to explore the possible binding interaction of the newly designed compounds with the key amino acid residues in the ATP binding site (Table 8). An X-ray structure of the active site of Aurora A kinase enzyme with embedded reference ligand (**II**, phthalazinone derivative) was used in this study (PDB ID: 3P9J) [15]. An initial docking experiment was conducted for the reference ligand (**II**) in the Aurora A kinase active site to validate the selected molecular docking protocol. The results of the validation step showed that the reference ligand (**II**) exhibited the same binding mode as reported in the protein data bank [15] with 100% pose occupancy (Figure 6A) with a docking score of −5.34118 kcal/mol. In addition, it revealed that the pyrazole ring occupied the hinge region and interacted with the key amino acid residues at this binding region (Glu 211, Tyr 212 and Ala 213). Moreover, the phthalazinone ring was anchored to the external region near the solvent-exposed area to bind with key aromatic amino acid residue in that region (Leu 139). At the same time, the terminal phenyl ring was pointed towards the solvent-exposed area with no additional visible binding interaction.

The tested compounds were then docked into the same prepared binding site to explore the possible virtual binding interactions. The molecular docking results showed that the tested compounds exhibited higher docking scores than the reference ligand. It is proposed that the employment of the quinazoline ring into the active site will enhance binding to the Aurora A kinase active site. However, in the case of reference ligand (**II**), the quinazoline ring was not fully fitted into the active site and stayed at the gate of the binding site instead. In addition, most of the generated poses of tested compounds shared the same binding pattern. Compound **6e** (the most active compound among the tested series in the in vitro kinase assay and cytotoxicity screening) showed an interesting new binding mode (70% pose occupancy) with key amino acid residues in the hinge region of the active site (Val 147, Glu 211, Tyr 212, Ala 213 and Leu 263) with both hydrophobic and halogen interactions (Table 9 and Figure 6B). Moreover, the quinazoline binding motif occupied the hinge region. The Br-substituted terminal phenyl ring engaged the external pocket with additional halogen and hydrophobic interactions. Furthermore, the free carboxylic group was pointed out and waved in the solvent-exposed area. Regarding ester-based derivatives (**5b** and **7a**,**b**), it was interesting to identify a new binding mode; the compound was flipped in order to anchor the ester group into the binding pocket rather than the solvent area (60% pose occupancy).

The docking results revealed that the fluorine group at the quinazoline ring has a significant impact on binding to the hinge region through additional binding along with the small-sized volume of this halogen atom (similar to a hydrogen atom) which prevents any clashes in that crucial region. In addition, the substitution of the terminal phenyl ring with a halogen atom (Br) modulated the orientation of this terminal ring through additional binding to the key amino acid residue in this area (Pro 214).

From the previous results (in vitro assay and in silico screening), the new quinazoline derivatives provide a promising selective inhibition profile toward Aurora A kinase. In addition, it delivers a new challenge to medicinal chemists to improve structural modification in order to enhance the inhibitory effect and maintain the selectivity profile. Moreover, enhancing the cell permeability is considered a key role in the same field to ensure cell induction and inhibition of the intracellular kinase (Aurora A) without any disruption in ATP competitive inhibition effect, as discussed before.

## 3. Materials and Methods

### 3.1. Chemistry

#### 3.1.1. General

All solvents and reagents were obtained from commercial suppliers and used without further purification. TLC was performed using glass sheets pre-coated with silica gel 60 F254 purchased by Merk. The NMR spectra were obtained on Bruker Avance 400. Column chromatography was carried out on Merck Silica Gel 60 (230–400 mesh), as previously described [16].

#### 3.1.2. General Procedure for the Synthesis of Derivatives **3a**–**e**

A solution of appropriate acid chloride (**2a**–**d**, 1 mmol, 1 Eq) in DCM was added dropwise to a solution of appropriate aromatic amine (**1a**,**b**, 1 mmol, 1 Eq) and triethylamine (1 mmol, 1 Eq) in DCM at 0 °C. The reaction mixture was stirred at rt for 4 h or until the TLC showed reaction completion. The organic layer was washed with brine (2 × 5 mL), dried over anhydrous Na_2_SO_4_ and concentrated under reduced pressure. The crude residue was purified by column chromatography to give the titled product **3a**–**e** [17].

#### 3.1.3. General Procedure for the Synthesis of Derivatives **4a**–**e**

A mixture of compound **3a**–**e** (1 mmol) and thionyl chloride (2 mL) was refluxed for 3 h. The excess thionyl chloride was removed under reduced pressure to obtain the titled product **4a**–**e** to be used in the next step without further purification [18].

#### 3.1.4. General Procedure for the Synthesis of Derivatives **5a**–**e**

Ethyl cyanoformate (1 mmol, 1 Eq) and tin tetrachloride (1 mmol, 1 Eq) were added to a solution of the appropriate benzanilide imidoyl chloride derivative (**4a**–**e**, 1 mmol, 1 Eq) in *o*-dichlorobenzene (1 mL). The reaction mixture was allowed to stir at 140 °C for 15 min. The reaction mixture was cooled to rt and produced alkaline with a cold 0 °C aqueous solution of NaOH (1 M). The mixture was then extracted with DCM, dried over magnesium sulfate and evaporated under reduced pressure. The crude residues were used directly in the next step without further purification except for compound **5b,** which was purified using column chromatography (2% EtOAc in hexane) [19].

*Ethyl 2-(3-chlorophenyl)quinazoline-4-carboxylate* (**5b**)

Grey solid, yield: 81%, mp: 75.2–76.3 °C, HPLC purity: 25.28 min, 98.39%, ^1^H NMR (400 MHz, CDCl_3_): *δ* 8.65 (s, 1H), 8.55–8.53 (m, 1H), 8.47 (d, *J* = 8.4 Hz, 1H), 8.16 (d, *J* = 8.5 Hz, 1H), 7.95 (t, *J* = 7.9 Hz, 1H), 7.68 (t, *J* = 7.9 Hz, 1H), 7.48–7.45 (m, 2H), 4.65 (q, *J* = 7.2 Hz, 2H), 1.54 (t, *J* = 7.2 Hz, 3H). ^13^C NMR (100 MHz, CDCl_3_) *δ* 165.10, 158.89, 157.84, 152.31, 139.21, 134.81, 134.61, 130.86, 129.88, 129.27, 128.74, 128.58, 126.83, 125.91, 120.55, 62.69, 14.30. HRMS (ESI) *m*/*z* calculated for C_17_H_13_ClN_2_O_2_ [M + H]^+^: 313.0744. Found: 313.0732.

#### 3.1.5. General Procedure for the Synthesis of Derivatives **6a**–**e**

A solution of corresponding ethyl esters (**5a**–**e**, 1 mmol, 1 Eq) in EtOH was stirred with an aqueous NaOH solution (20%, 5 mL). The reaction mixture was refluxed for 2 h. The reaction mixture was concentrated under reduced pressure, acidified with a citric acid solution (20%, 5 mL) and extracted with EtOAc (10 mL). The organic layer was washed with brine (2 × 5 mL), dried over anhydrous Na_2_SO_4_ and concentrated under reduced pressure to give the titled product **6a**–**e** [19].

*2-Phenylquinazoline-4-carboxylic acid* **(6a)**

Brown solid, yield: 31%, mp: 146.2–147.2 °C, HPLC purity: 14.20 min, 97.79%, ^1^H NMR (400 MHz, DMSO-*d*_6_): *δ* 8.59–8.57 (m, 2H), 8.42 (d, *J* = 8.3 Hz, 1H), 8.18–8.09 (m, 2H), 7.81 (t, *J* = 7.9 Hz, 1H), 7.61–7.59 (m, 3H).

*2-(3-Chlorophenyl)quinazoline-4-carboxylic acid* (**6b**)

Yellow solid, yield: 86%, mp: 175.6–176.6 °C, HPLC purity: 18.41 min, 99.93%, ^1^H NMR (400 MHz, DMSO-*d_6_*): *δ* 8.55 (s, 1H), 8.52 (d, *J* = 6.9 Hz, 1H), 8.46 (d, *J* = 8.3 Hz, 1H), 8.19–8.09 (m, 2H), 7.83 (t, *J* = 7.6 Hz, 1H), 7.65–7.61 (m, 2H). HRMS (ESI) *m*/*z* calculated for C_15_H_9_ClN_2_O_2_ [M + H]^+^: 285.0431. Found: 285.0419.

*8-Fluoro-2-(m-tolyl)quinazoline-4-carboxylic acid* (**6c**)

White solid, yield: 5%, mp: 349.3–350.3 °C, HPLC purity: 17.40 min, 97.03%, ^1^H NMR (400 MHz, DMSO-*d*_6_): *δ* 8.38–8.33 (m, 2H), 7.98 (d, *J* = 8.2 Hz, 1H), 7.79–7.75 (m, 1H), 7.62–7.56 (m, 1H), 7.48–7.44 (m, 1H), 7.39–7.37 (m, 1H), 2.46 (s, 3H). ^13^C NMR (100 MHz, DMSO-*d*_6_) *δ* 170.02, 168.67, 160.17, 158.21, 155.67, 141.00, 138.25, 137.97, 131.97, 129.12, 127.00, 125.90, 124.14, 121.02, 118.16, 21.59. HRMS (ESI) *m*/*z* calculated for C_16_H_11_FN_2_O_2_ [M + H]^+^: 283.0883. Found: 283.0871.

*8-Fluoro-2-phenylquinazoline-4-carboxylic acid* (**6d**)

White solid, yield: 7%, mp: 353.3–354.3 °C, HPLC purity: 15.33 min, 99.79%, ^1^H NMR (400 MHz, DMSO-*d*_6_): *δ* 8.55 (s, 2H), 7.99 (s, 1H), 7.79–7.58 (m, 4H). HRMS (ESI) *m*/*z* calculated for C_15_H_9_FN_2_O_2_ [M + H]^+^: 269.0726. Found: 269.0714.

*2-(3-Bromophenyl)-8-fluoroquinazoline-4-carboxylic acid* (**6e**)

White solid, yield: 4%, mp: 335.4–336.4 °C, HPLC purity: 19.74 min, 99.86%, ^1^H NMR (400 MHz, DMSO-*d*_6_): *δ* 8.66 (s, 1H), 8.54 (d, *J* = 7.8 Hz, 1H), 8.01 (d, *J* = 8.2 Hz, 1H), 7.83–7.76 (m, 2H), 7.66–7.54 (m, 2H).

#### 3.1.6. General Procedure for the Synthesis of Derivatives **7a**,**b**

Sulfuric acid (0.2 mL) was added to a solution of compound **6b** (1 mmol) in appropriate alcohol (10 mL). The reaction mixture was refluxed for 18 h. The reaction mixture was cooled, quenched with a saturated solution of NaHCO_3_ (2 mL) and concentrated under reduced pressure. The crude residue was extracted between EtOAc (10 mL) and water (5 mL). The organic layer was washed with brine (2 × 5 mL), dried over anhydrous Na_2_SO_4_ and concentrated under reduced pressure to give the titled product **7a**,**b**.

*Methyl 2-(3-chlorophenyl)quinazoline-4-carboxylate* (**7a**)

White solid, yield: 91%, mp: 124.4–125.4 °C, HPLC purity: 23.52 min, 99.96%, ^1^H NMR (400 MHz, CDCl_3_): *δ* 8.64 (s, 1H), 8.54–8.50 (m, 2H), 8.16 (d, *J* = 8.5 Hz, 1H), 7.98–7.93 (m, 1H), 7.70–7.66 (m, 1H) 7.50–7.44 (m, 2H) 4.15 (s, 3H). ^13^C NMR (100 MHz, CDCl_3_) *δ* 165.46, 158.88, 157.17, 152.41, 139.15, 134.85, 134.67, 130.89, 129.92, 129.28, 128.73, 128.70, 126.80, 125.97, 120.66, 53.32. HRMS (ESI) *m*/*z* calculated for C_16_H_11_ClN_2_O_2_ [M + H]^+^: 299.0587. Found: 299.0575.

*Isopropyl 2-(3-chlorophenyl)quinazoline-4-carboxylate* (**7b**)

Greenish grey solid, yield: 89%, mp: 72.6–73.6 °C, HPLC purity: 26.90 min, 96.79%, ^1^H NMR (400 MHz, CDCl_3_): *δ* 8.65 (s, 1H), 8.59–8.53 (m, 1H), 8.39 (d, *J* = 8.4 Hz, 1H), 8.14 (d, *J* = 8.5 Hz, 1H), 7.96–7.92 (m, 1H), 7.68–7.64 (m, 1H), 7.49–7.44 (m, 2H), 5.56–5.46 (m, 1H), 1.54 (d, *J* = 6.3 Hz, 6H). ^13^C NMR (100 MHz, CDCl_3_) *δ* 164.74, 158.89, 158.48, 152.21, 139.24, 134.77, 134.53, 130.83, 129.85, 129.26, 128.74, 128.46, 126.85, 125.79, 120.42, 70.81, 21.92. HRMS (ESI) *m*/*z* calculated for C_18_H_15_ClN_2_O_2_ [M + H]^+^: 327.0900. Found: 327.0887.

### 3.2. In Vitro Screening

#### 3.2.1. Kinase Inhibitory Assay

The in vitro kinase inhibitory assay was performed at Reaction Biology Corp (http://www.reactionbiology.com accessed on 15 February 2022) using Kinase HotSpotSM in order to measure the inhibitory activities of tested candidates, as previously performed [20,21]. The experiment contained specific kinase/substrate pairs along with required cofactors. Base reaction buffer: 20 mM Hepes (pH 7.5), 10 mM MgCl_2_, 1 mM EGTA, 0.02% Brij35, 0.02 mg/mL BSA, 0.1 mM Na_3_VO_4_, 2 mM DTT, 1% DMSO. The tested compounds were dissolved in 100% DMSO to a specific concentration. The serial dilution was conducted by Integra Viaflo Assist in DMSO. The reaction mixture containing the examined compound and 33P-ATP was incubated at rt for 2 h, and radioactivity was detected by the filter-binding method. Kinase activity data were expressed as the percentage of remaining kinase activity in test samples compared to vehicle (DMSO) reactions.

#### 3.2.2. Cytotoxicity Assay

The in vitro cytotoxic screening of tested compounds (**5b**, **6a**–**e** and **7a**,**b**) over nine NCI human cancer cell lines was carried out at the National Cancer Institute (NCI), Bethesda, Maryland, USA (dtp.cancer.gov accessed on 25 February 2022), as previously performed [22]. A 48 h drug exposure protocol was adopted, and a sulforhodamine B (SRB) assay was utilized to assess the cell growth and viability. The human tumor cell lines of the cancer screening panel were grown in RPMI 1640 medium containing 5% fetal bovine serum and 2 mM L-glutamine. For a typical screening experiment, cells were inoculated into 96 well microtiter plates in 100 µL at plating densities ranging from 5000 to 40,000 cells/well, depending on the doubling time of individual cell lines. After cell inoculation, the microtiter plates were incubated at 37 °C, 5% CO_2_, 95% air and 100% relative humidity for 24 h prior to the addition of experimental drugs.

After 24 h, two plates of each cell line were fixed in situ with TCA to represent a measurement of the cell population for each cell line at the time of drug addition (Tz). Experimental compounds were solubilized in dimethylsulfoxide at 400-fold the desired final maximum test concentration and stored frozen prior to use. At the time of compound addition, an aliquot of frozen concentrate was thawed and diluted to twice the desired final maximum test concentration with a complete medium containing 50 µg/mL gentamicin. Aliquots of 100 µL were added to the appropriate microtiter wells already containing 100 µL of the medium, resulting in the required final compound concentration (10 µM).

Following test compound addition, the plates were incubated for an additional 48 h at 37 °C, 5% CO_2_, 95% air and 100% relative humidity. For adherent cells, the assay was terminated by the addition of cold TCA. Cells were fixed in situ by the gentle addition of 50 µL of cold 50% (*w*/*v*) TCA (final concentration, 10% TCA) and incubated for 60 min at 4 °C. The supernatant was discarded, and the plates were washed five times with tap water and air-dried. Sulforhodamine B (SRB) solution (100 µL) at 0.4% (*w*/*v*) in 1% acetic acid was added to each well, and plates were incubated for 10 min at rt. After staining, the unbound dye was removed by washing five times with 1% acetic acid, and the plates were air-dried. The bound stain was subsequently solubilized with 10 mM trizma base, and the absorbance was read on an automated plate reader at a wavelength of 515 nm. For suspension cells, the methodology is the same, except that the assay was terminated by fixing settled cells at the bottom of the wells by gently adding 50 µL of 80% TCA (final concentration, 16% TCA).

The additional in vitro cytotoxicity cell assay for compound **6e** was carried out over urinary bladder (T-24) and breast cancer (MCF-7) cell lines to evaluate IC_50_. Both cell lines were obtained from Nawah Scientific Inc. (Mokatam, Cairo, Egypt). T-24 and MCF-7 cells were maintained in McCoy’s and DMEM, respectively, and supplemented with 100 mg/mL of streptomycin, 100 units/mL of penicillin and 10% of heat-inactivated fetal bovine serum in a humidified, 5% (*v*/*v*) CO_2_ atmosphere at 37 °C. Cell viability was assessed by an SRB assay. Aliquots of 100 μL cell suspension (5 × 10^3^ cells) were placed in 96-well plates and incubated in complete media for 24 h. Cells were treated with another aliquot of 100 μL media containing compound **6e** at various concentrations (0.1, 1, 10, 100, 1000 μM as final concentrations). After 72 h of compound **6e** exposure, cells were fixed by replacing the media with 150 μL of 10% TCA and incubated at 4 °C for 1 h. The TCA solution was removed, and the cells were washed five times with distilled water. Aliquots of 70 μL SRB solution (0.4% *w*/*v*) were added and incubated in a dark place at rt for 10 min. Plates were washed three times with 1% acetic acid and allowed to air-dry overnight. Then, 150 μL of TRIS (10 mM) was added to dissolve the protein-bound SRB stain; the absorbance was measured at 540 nm using a BMG LABTECH^®^-FLUOstar Omega microplate reader (Ortenberg, Germany) [23,24].

#### 3.2.3. Cell Cycle

Cell cycle distribution was measured after the treatment of MCF-7 cells with compound **6e** (at its IC_50_ value over MCF-7 cell line) and paclitaxel (1 µM) for 24 h as a positive control. Cells (10^5^ cells) were collected by trypsinization and washed twice with ice-cold PBS (pH 7.4). Cells were re-suspended in 2 mL of 60% ice-cold EtOH and incubated at 4 °C for 1 h for fixation. Fixed cells were washed twice again with PBS (pH 7.4) and re-suspended in 1 mL of PBS containing 50 µg/mL RNAase A and 10 µg/mL propidium iodide (PI). After 20 min of incubation in the dark at 37 C, cells were analyzed for DNA contents using flow cytometry analysis using FL2 (λ_ex/em_ 535/617 nm) signal detector (ACEA Novocyte™ flow cytometer, ACEA Biosciences Inc., San Diego, CA, USA). Twelve thousand events were acquired. Cell cycle distribution was calculated using ACEA NovoExpress™ software (ACEA Biosciences Inc., San Diego, CA, USA) [24,25,26,27,28].

#### 3.2.4. Apoptosis

Apoptosis and necrosis cell populations were determined using the Annexin V-FITC apoptosis detection kit (Abcam Inc., Cambridge Science Park, Cambridge, UK) coupled with 2 fluorescent channels flowcytometry. After treatment with compound **6e** (at its IC_50_ value over MCF-7 cell line) and doxorubicin (10 µM) for 24 h as a positive control, MCF-7 cells (10^5^ cells) were collected by trypsinization and washed twice with ice-cold PBS (pH 7.4). Then, cells were incubated in the dark with 0.5 mL of Annexin V-FITC/PI solution for 30 min at rt according to the manufacturer’s protocol. After staining, cells were injected via an ACEA Novocyte™ flow cytometer (ACEA Biosciences Inc., San Diego, CA, USA) and analyzed for FITC and PI fluorescent signals using FL1 and FL2 signal detectors, respectively (λ_ex/em_ 488/530 nm for FITC and λ_ex/em_ 535/617 nm for PI). Twelve thousand events were acquired, and positive FITC and PI cells were quantified by quadrant analysis and calculated using ACEA NovoExpress™ software (ACEA Biosciences Inc., San Diego, CA, USA) [25,27,28].

### 3.3. In Silico Screening

A molecular docking study was conducted using Discovery Studio Client 21.1.0.20298 (Biovia Corp) (www.3ds.com accessed on 27 February 2022). The X-ray structure of the kinase domain of Aurora A kinase enzyme was downloaded from the protein data bank (www.rcsb.org accessed on 28 February 2022) (PDB ID: 3P9J) [15]. Water molecules were removed, keeping only the kinase domain with its corresponding reference ligand (compound **II**). The bonds and bond orders of the amino acid chain were checked and corrected. The terminal residues were checked and adjusted. 

A CHARMm forcefield was applied to the protein domain. A Momany–Rone forcefield was selected for the partial charge. The binding site was defined using the coordinates of the reference ligand. The CDOCKER algorithm (CHARMm-based molecular dynamic scheme) [29] was used in this study to generate the most stable conformers with the binding sites. Random ligand conformations were generated (10 conformers) from the initial ligand structure through dynamic target temperature (100 K), followed by random rotations. The generated conformers were refined by grid-based (GRID-1) simulated annealing.

The docking protocol was validated by running an initial docking experiment (pre-docking) for the reference ligand (**II**, PDB ID: P9J). The docking experiment of compound **6e** was conducted to generate the 10 most possible conformers with the binding site. The generated conformers were visualized to investigate the binding interaction between the tested compound (**6e**) and key amino acid residues in the active site of the Aurora A kinase domain.

## 4. Conclusions

A number of quinazoline-based derivatives were designed and synthesized based on the structural modification of previously reported Aurora A kinase inhibitors (**I**–**III**). Free carboxylic acid-bearing derivatives (**6a**–**e**) as well as ester-based compounds (**5b** and **7a**,**b**) were tested for their inhibitory activities over the Aurora A kinase enzyme. Compound **6e** exhibited the most potent inhibitory activity among the tested compounds. Furthermore, selectivity profile testing was conducted for compound **6e** over 14 kinases (including Aurora B kinase). In addition, an in vitro cytotoxicity assay was conducted against NCI human cancer cell lines. The CNS cancer cell line (SNB-75) was the most sensitive cell line among the tested lines. Both cell cycle and cellular apoptosis analyses were carried out to compound **6e**. Compound **6e** was able to arrest the cell cycle of the MCF-7 cell line at the G1 phase. In addition, it induced apoptosis compared to the positive control. In silico molecular docking screening was carried out to explore the binding mode of compound **6e** into the active site of the Aurora A kinase domain. It exhibited perfect fitting into the active side key regions compared to the reference ligand (compound **II**). In addition, it showed potential binding interactions with the key amino acid residues in the kinase pocket. The results revealed that both halogens in the central core scaffold and terminal phenyl ring have a significant role in the binding to the active site. Furthermore, the small-sized halogen atom (F) introduced into the central core had the perfect size to avoid any clashes and maintain the perfect fitting into the active site. In conclusion, the new quinazoline derivatives can be considered a potential hit for further structural and molecular optimization in future.

## Figures and Tables

**Table 1:**
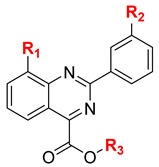
Key structure of target compounds.

Compound	R_1_	R_2_	R_3_
**5b**	H	Cl	Et
**6a**	H	H	H
**6b**	H	Cl	H
**6c**	F	Me	H
**6d**	F	H	H
**6e**	F	Br	H
**7a**	H	Cl	Me
**7b**	H	Cl	*i*-Pr

**Table 2:** In vitro kinase inhibitory assay (inhibition %) of free carboxylic acid-based derivatives (**6a**–**e**) over Aurora A kinase.

Compound	Aurora A _(Inhibition%)_
**6a**	7.64 ± 1.19
**6b**	28.48 ± 2.40
**6c**	14.83 ± 0.14
**6d**	22.18 ± 1.86
**6e**	51.78 ± 0.38

**Table 3:** In vitro kinase inhibitory assay (inhibition%) of ester-based derivatives (**5b** and **7a**,**b**) over Aurora A kinase.

Compound	Aurora A _(Inhibition%)_
**5b**	6.53 ± 0.58
**7a**	−2.61 ± 0.15
**7b**	3.83 ± 1.38

**Table 4:** In vitro kinase inhibitory assay (activity%) of compound **6e** over kinase panel.

Kinase	Compound 6e _(Activity%)_	Kinase	Compound 6e _(Activity%)_
ABL-1	106.77	CDK-2/cyclin A	83.72
AKT-1	104.50	CDK-8/cyclin C	97.81
ALK	99.86	BRAFV599E	100.92
Aurora A	48.22	RAF-1	111.64
Aurora B	92.90	MAPK APK-2	99.55
CAMKK-2	83.66	MEK-1	85.42
VRK-2	118.72	mTOR/FRAP-1	105.00

**Table 5:** In vitro cytotoxicity assay (growth%) of compounds **5b**, **6a**–**e** and **7a**,**b** over NCI human CNS cancer cell line (SNB-75).

Compound	SNB-75 _(Growth%)_	Compound	SNB-75 _(Growth%)_
**5b**	75.14	**6d**	82.18
**6a**	88.17	**6e**	66.99
**6b**	73.59	**7a**	80.12
**6c**	79.79	**7b**	75.14

**Table 6:** In vitro cell cycle assay results of compound **6e** in MCF-7 cell line.

Cell Cycle Phase	Positive Control _(%)_	Compound 6e _(%)_
G1	51.45	60.68
S	22.79	17.47
G2	21.34	18.29

**Table 7:** In vitro cellular apoptosis assay of compound **6e** in MCF-7 cell line.

Viability	Positive Control (%)	Compound 6e (%)
Intact cells	98.48	97.83
Early apoptosis	0.08	0.1
Late apoptosis	0.68	0.81
Necrosis	0.76	1.26
Total death	1.52	2.16

**Table 8:** In silico molecular docking score (kcal/mol) of the tested compounds (**5b**, **6a**–**e** and **7a**,**b**) into Aurora A kinase domain (PDB ID: 3P9J).

Compound	Docking Score _(kcal/mol)_	Compound	Docking Score _(kcal/mol)_
**5b**	−11.9746	**6d**	−13.4928
**6a**	−11.0161	**6e**	−14.0872
**6b**	−12.5924	**7a**	−12.6606
**6c**	−16.2153	**7b**	−11.3566

**Table 9:** Possible binding interactions of compound **6e** into Aurora A active site.

Binding Motif	Amino Acid *	Binding Interaction	Distance (Å)
Central motif (quinazoline)			
Phenyl	Ala 160 Val 147 Leu 263	Hydrophobic Hydrophobic Hydrophobic	- - -
Pyrimidine	Leu 139 Val 147 Leu 263	Hydrophobic Hydrophobic Hydrophobic	- - -
F	Glu 211 Tyr 212 Ala 213	Halogen Halogen Halogen	3.52 2.61 2.49
Terminal motif			
Phenyl	Leu 139	Hydrophobic	-
Br	Leu 139 Tyr 212 Pro 214	Halogen Halogen Halogen	- - 3.27

* Ala, alanine; Val, valine; Leu, leucine; Glu, glutamine; Tyr, tyrosine; Pro, proline.

## Data Availability

All of the data are contained within the article and the Supplementary Materials.

## Supplementary Materials

The following are available online at https://www.mdpi.com/2075-1729/12/6/876/s1, experimental and spectral analysis of both key intermediates and final target compounds. In vitro cytotoxicity data over NCI human cancer cell lines (Table S1–S8).
