# Correction: Elsherbeny et al. 2-(3-Bromophenyl)-8-fluoroquinazoline-4-carboxylic Acid as a Novel and Selective Aurora A Kinase Inhibitory Lead with Apoptosis Properties: Design, Synthesis, In Vitro and In Silico Biological Evaluation. *Life* 2022, *12*, 876

**DOI:** 10.3390/life14040423

**Published:** 2024-03-22

**Authors:** Mohamed H. Elsherbeny, Usama M. Ammar, Magda H. Abdellattif, Mohammed A. S. Abourehab, Ahmed Abdeen, Samah F. Ibrahim, Doaa Abdelrahaman, Wessam Mady, Eun Joo Roh, Ahmed Elkamhawy

**Affiliations:** 1 Chemical and Biological Integrative Research Center, Korea Institute of Science and Technology (KIST), Seoul 02792, Republic of Korea; mohamed.alsherbeny@pharma.asu.edu.eg; 2 Division of Bio-Medical Science & Technology, KIST School, University of Science and Technology, Seoul 02792, Republic of Korea; 3 Pharmaceutical Chemistry Department, Faculty of Pharmacy, Ahram Canadian University, Giza 12566, Egypt; 4 Strathclyde Institute of Pharmacy and Biomedical Sciences, University of Strathclyde, 161 Cathedral Street, Glasgow G4 0NR, UK; usama.ammar@strath.ac.uk; 5 Department of Chemistry, College of Science, Taif University, P.O. Box 11099, Taif 21944, Saudi Arabia; m.hasan@tu.edu.sa; 6 Department of Pharmaceutics, College of Pharmacy, Umm Al-Qura University, Makkah 21955, Saudi Arabia; maabourehab@uqu.edu.sa; 7 Department of Pharmaceutics and Industrial Pharmacy, College of Pharmacy, Minia University, Minia 61519, Egypt; 8 Department of Forensic Medicine and Toxicology, Faculty of Veterinary Medicine, Benha University, Toukh 13736, Egypt; ahmed.abdeen@fvtm.bu.edu.eg; 9 Department of Clinical Sciences, College of Medicine, Princess Nourah bint Abdulrahman University, P.O. Box 84428, Riyadh 11671, Saudi Arabia; sfibrahim@pnu.edu.sa (S.F.I.); dsabdelrahman@pnu.edu.sa (D.A.); 10 Center of Excellence in Genomic Medicine Research, King Abdulaziz University, Jeddah 21589, Saudi Arabia; wmadi@kau.edu.sa; 11 BK21 FOUR Team and Integrated Research Institute for Drug Development, College of Pharmacy, Dongguk University-Seoul, Goyang 10326, Republic of Korea; a_elkamhawy@mans.edu.eg; 12 Department of Pharmaceutical Organic Chemistry, Faculty of Pharmacy, Mansoura University, Mansoura 35516, Egypt

In the original publication [1], reference number 26 [2] was added by mistake. Thus, it was removed.

With this correction, the order of some references has been adjusted accordingly. The authors state that the scientific conclusions are unaffected. This correction was approved by the Academic Editor. The original publication has also been updated.
