# Supplementary material for: 2-(3-Bromophenyl)-8-fluoroquinazoline-4-carboxylic Acid as a Novel and Selective Aurora A Kinase Inhibitory Lead with Apoptosis Properties: Design, Synthesis, In Vitro and In Silico Biological Evaluation
Source: Life (Basel). 2022 Jun 10;12(6):876. doi: 10.3390/life12060876 (PMC9225547; doi:10.3390/life12060876)

2-(3-Bromophenyl)-8-fluoroquinazoline-4-carboxylic Acid as a Novel and Selective Aurora A Kinase Inhibitory Lead with Apoptosis Properties: Design, Synthesis, In Vitro and In Silico Biological Evaluation

Supplementary Materials

**Index**	**Page**
S.1. Chemistry	2
S1.1. ^{1}HNMR and ^{13}CNMR data.	2
S1.2. HPLC purity data.	8
S1.3. Representative HRMS data.	12
S2. *In vitro* screening	15

S1. Chemistry

S1.1. ¹HNMR and ¹³CNMR data.

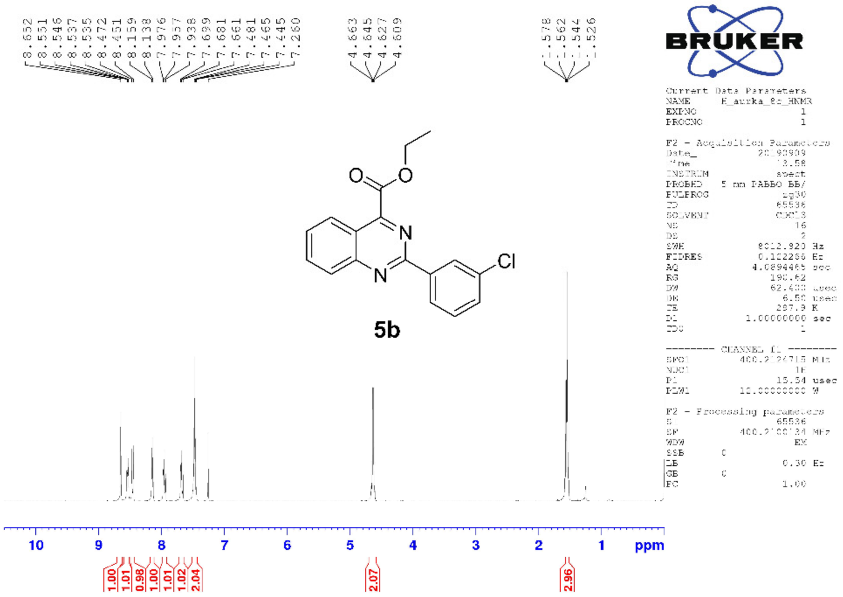

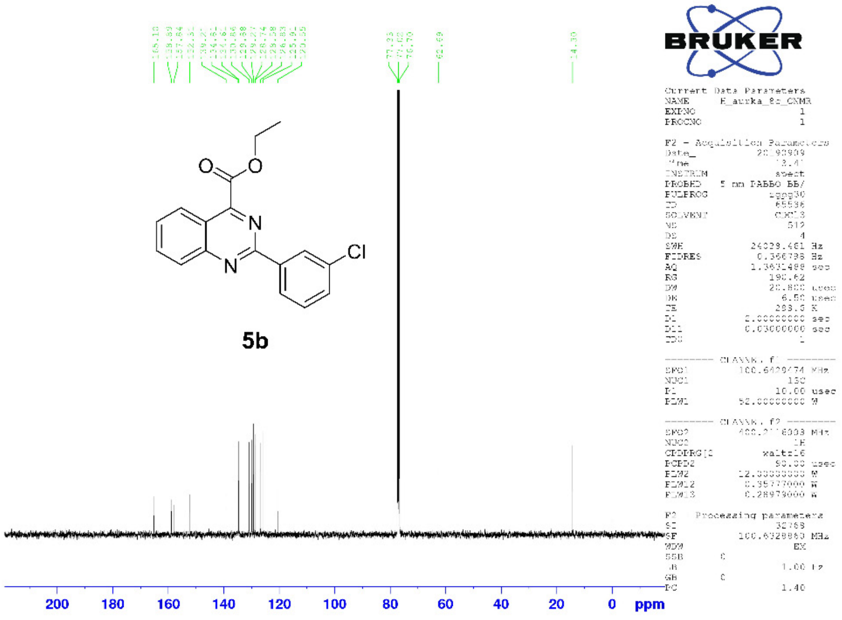

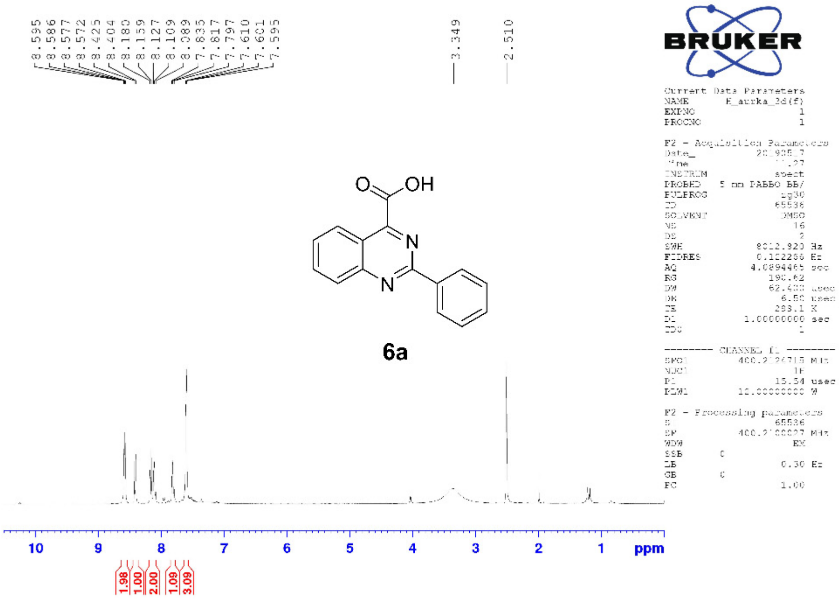
6a

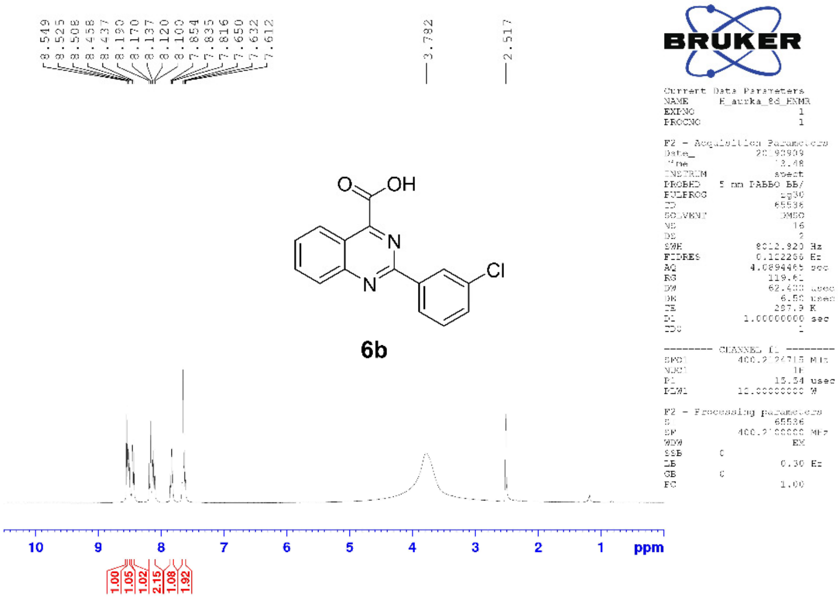
6b

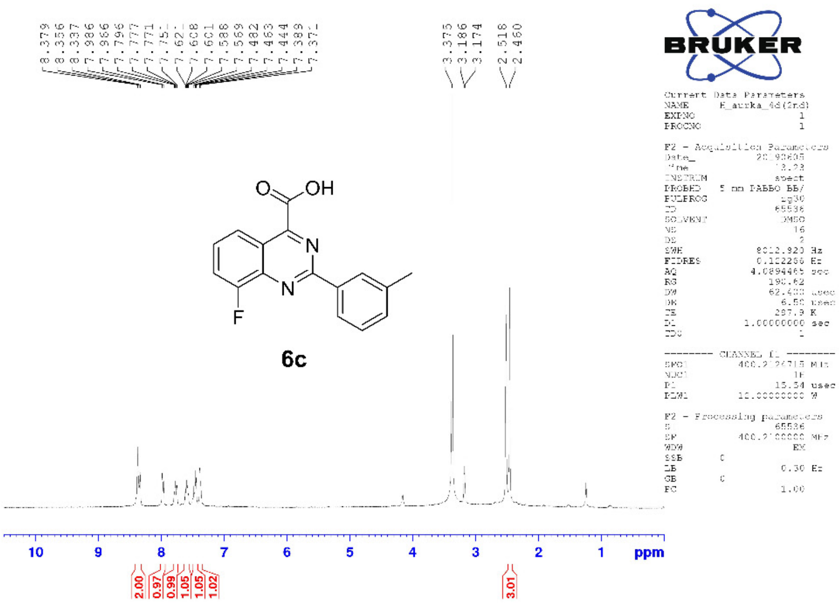

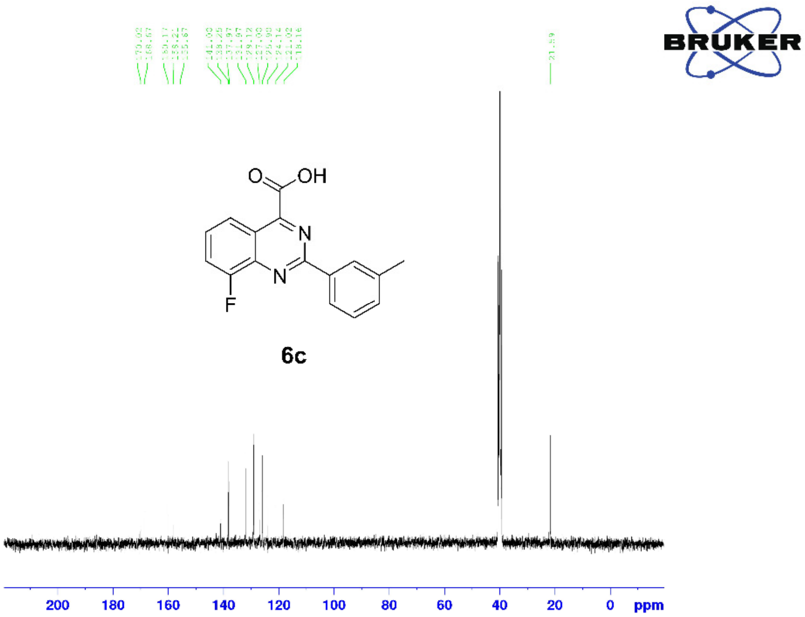

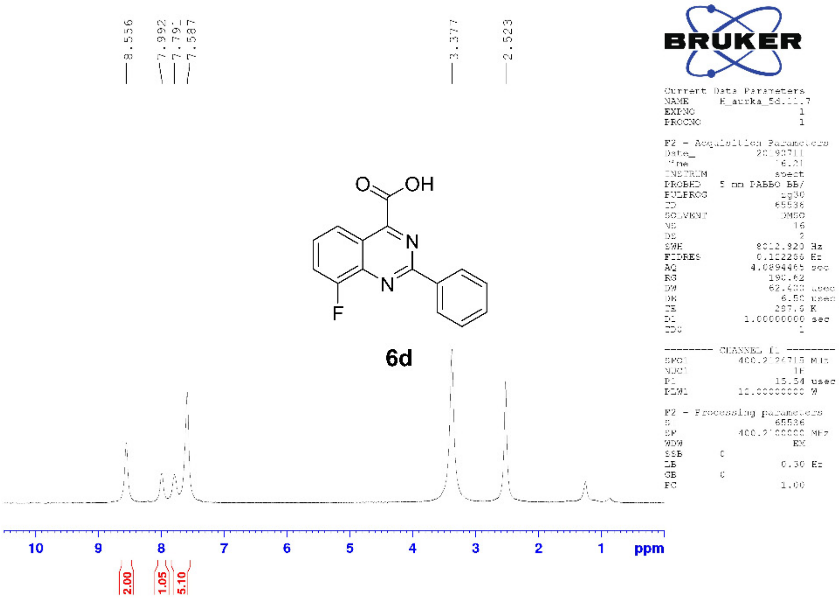
8.556
7.992
7.791
7.587
3.377
2.523
BRUKER
O
OH
N
N
F
10 9 8 7 6 5 4 3 2 1 ppm
2.00
1.05
5.10

6d

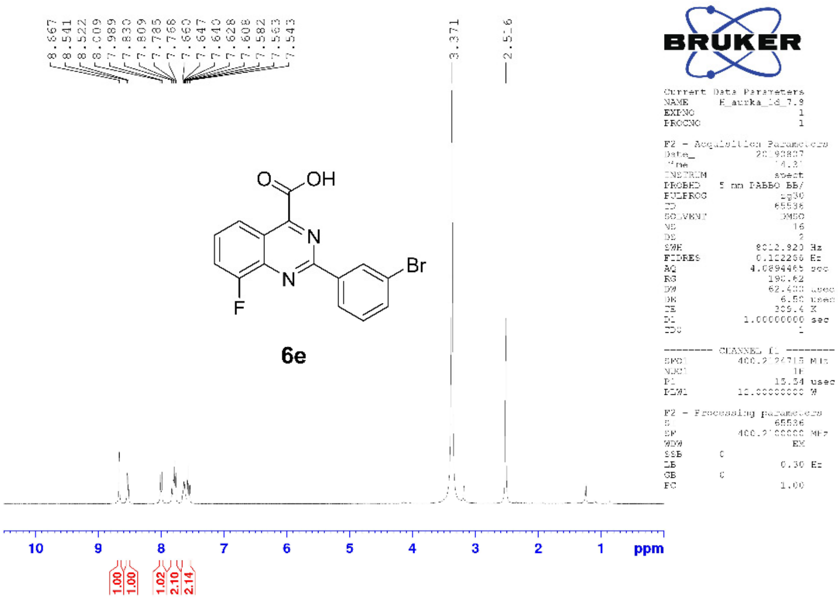
8.567
8.541
8.522
8.009
7.989
7.830
7.809
7.785
7.768
7.660
7.647
7.640
7.628
7.608
7.582
7.563
7.543
3.371
2.516
BRUKER
O
OH
N
N
Br
F
10 9 8 7 6 5 4 3 2 1 ppm
1.00
1.00
1.02
2.10
2.14

6e

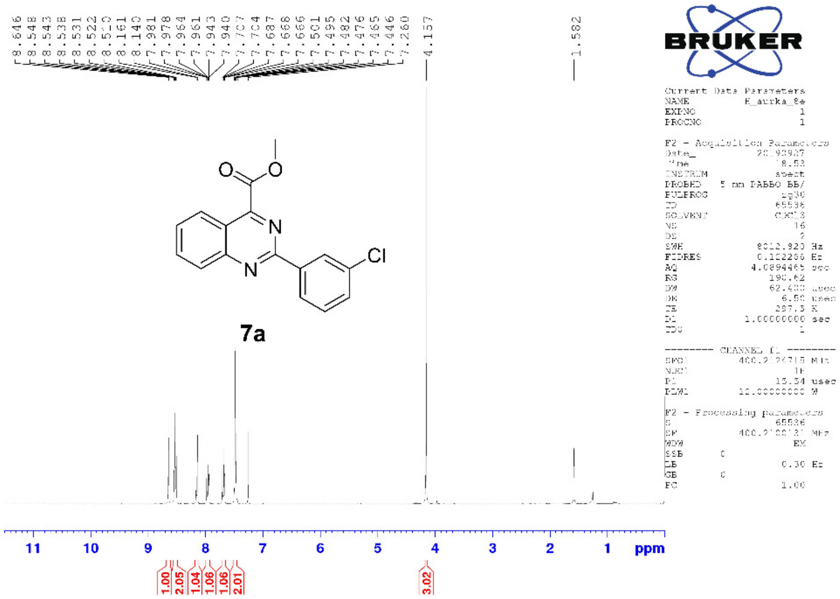
BRUKER
7a
ppm

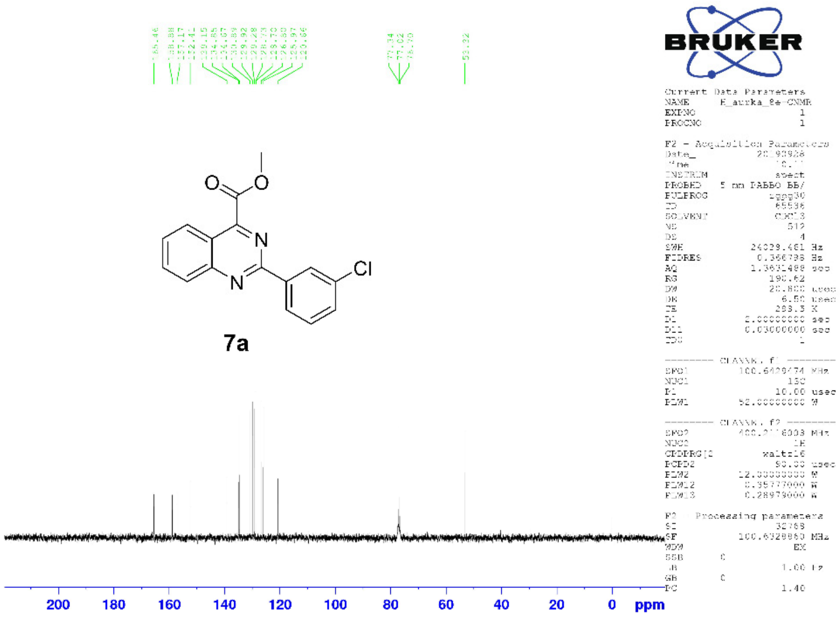
BRUKER
7a
ppm

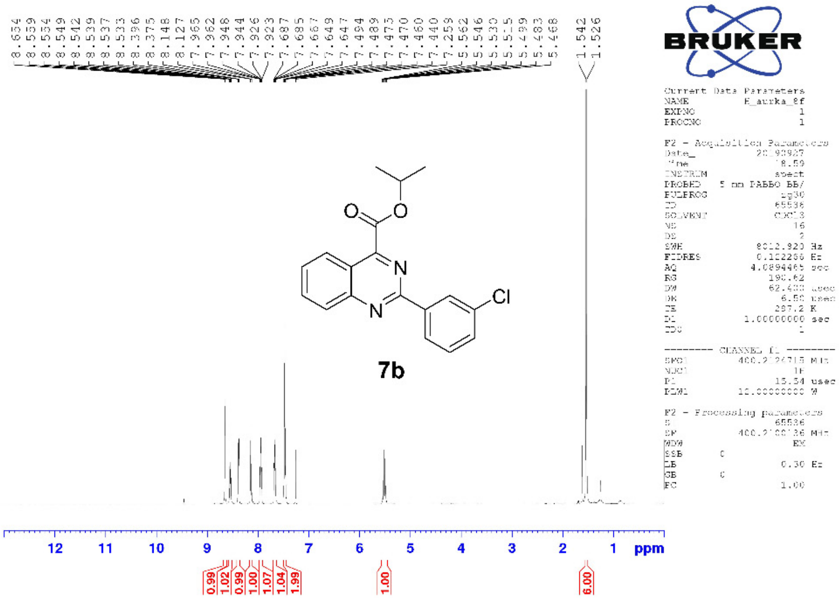

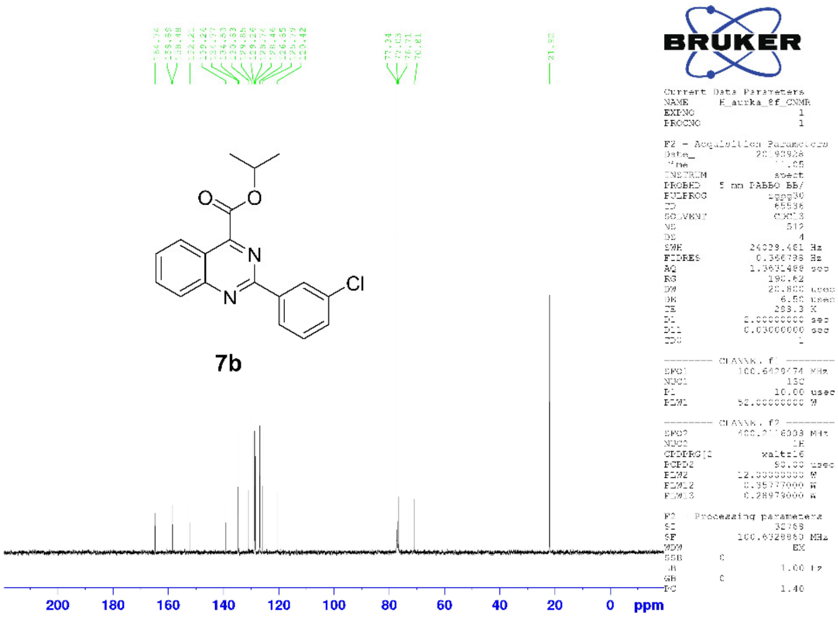

S1.2. HPLC purity data.

	Name	Retention Time (min)	Purity1 Angle	Purity1 Threshold	PDA Match1 Spect. Name	PDA Match1 Angle	PDA Match1 Threshold	PDA Match1 Lib. Name	Area (µV*sec)	% Area	Height (µV)	Int Type	Amount
1		23.453							359039	1.25	61587	bb	
2		25.281							28253726	98.39	2696270	bb	
3		27.274							102511	0.36	17051	bb	

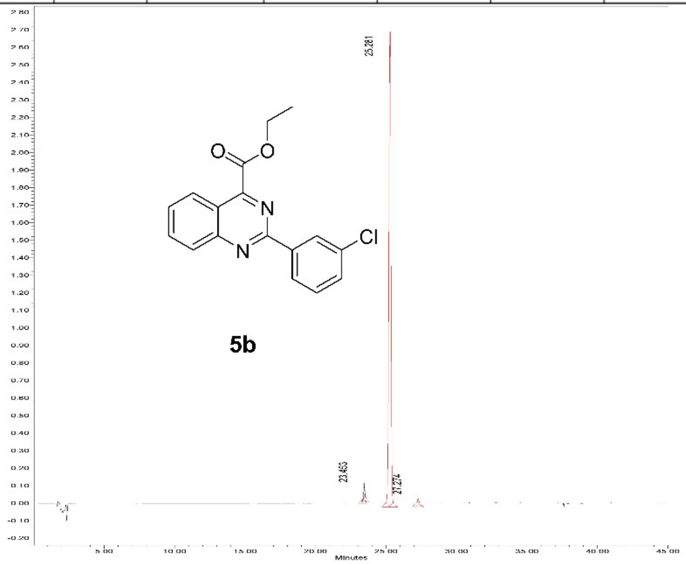

5b

	Name	Retention Time (min)	Purity1 Angle	Purity1 Threshold	PDA Match1 Spect. Name	PDA Match1 Angle	PDA Match1 Threshold	PDA Match1 Lib. Name	Area (µV*sec)	% Area	Height (µV)	Int Type	Amount
1		4.275							69216	0.43	19709	bb	
2		12.042							84239	0.52	16232	bb	
3		14.201							15812613	97.79	1723518	bb	
4		25.186							203671	1.26	30833	bb	

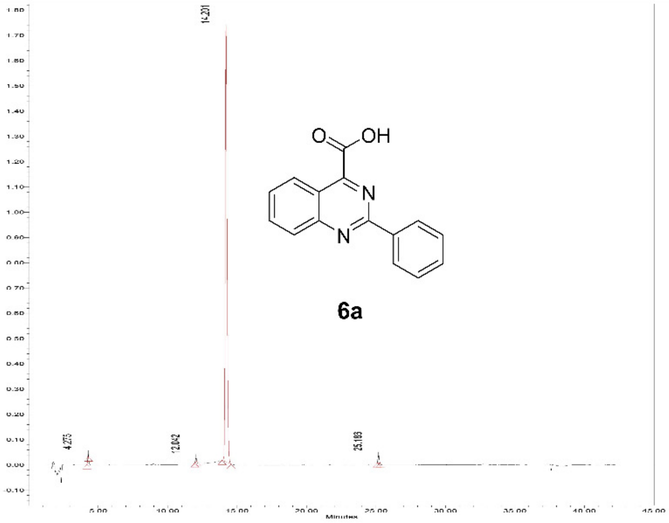

6a

	Name	Retention Time (min)	Purity1 Angle	Purity1 Threshold	PDA Match1 Spect. Name	PDA Match1 Angle	PDA Match1 Threshold	PDA Match1 Lib. Name	Area (µV*sec)	% Area	Height (µV)	Int Type	Amount
1		18.414							19695683	99.93	1917425	bb	
2		22.068							13558	0.07	1639	bb	

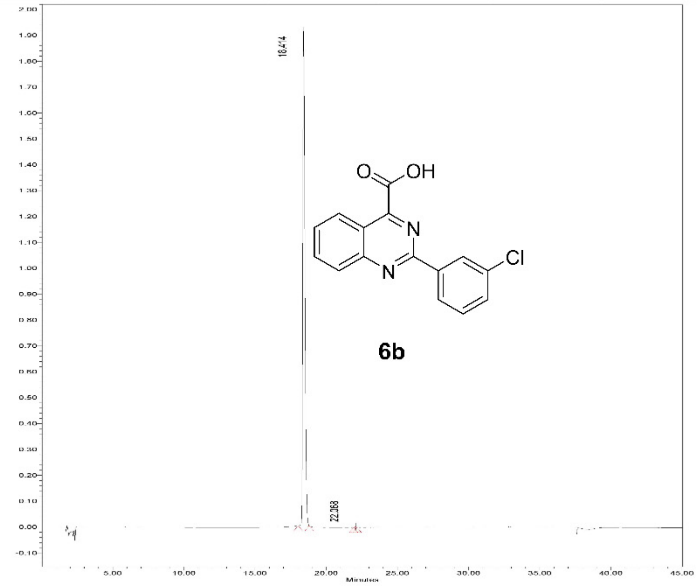

6b

	Name	Retention Time (min)	Purity1 Angle	Purity1 Threshold	PDA Match1 Spect. Name	PDA Match1 Angle	PDA Match1 Threshold	PDA Match1 Lib. Name	Area (µV*sec)	% Area	Height (µV)	Int Type	Amount
1		15.330							25297	0.15	4601	bb	
2		16.610							18741	0.11	3390	bb	
3		16.889							199424	1.19	29974	bb	
4		17.398							16311815	97.03	1591978	bb	
5		20.196							256282	1.52	37135	bb	

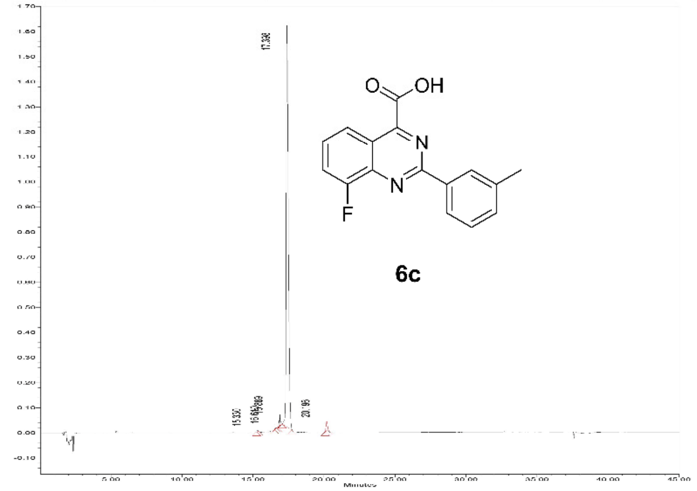

6c

	Name	Retention Time (min)	Purity1 Angle	Purity1 Threshold	PDA Match1 Spect. Name	PDA Match1 Angle	PDA Match1 Threshold	PDA Match1 Lib. Name	Area (µV*sec)	% Area	Height (µV)	Int Type	Amount	Units
1		11.027							5965	0.21	1155	bb		
2		15.328							2839909	99.79	303738	bb		

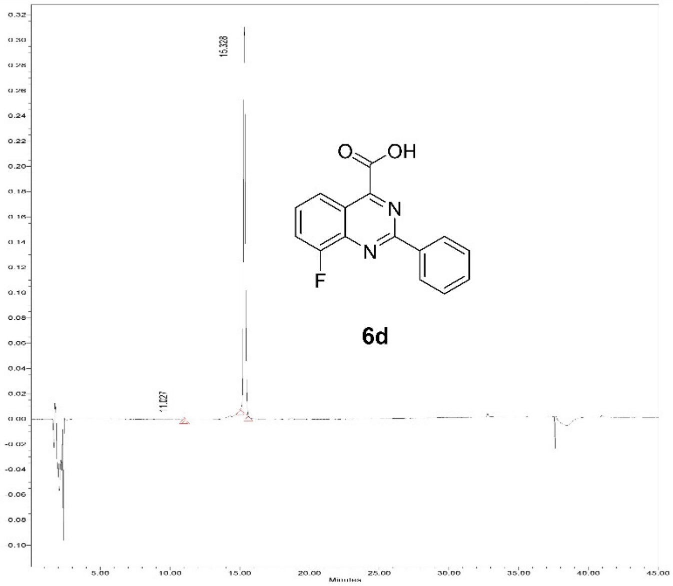

	Name	Retention Time (min)	Purity1 Angle	Purity1 Threshold	PDA Match1 Spect. Name	PDA Match1 Angle	PDA Match1 Threshold	PDA Match1 Lib. Name	Area (µV*sec)	% Area	Height (µV)	Int Type	Amount	Units
1		18.814							4641	0.05	1212	bb		
2		19.205							7994	0.09	1605	bb		
3		19.742							8817287	99.86	786244	bb		

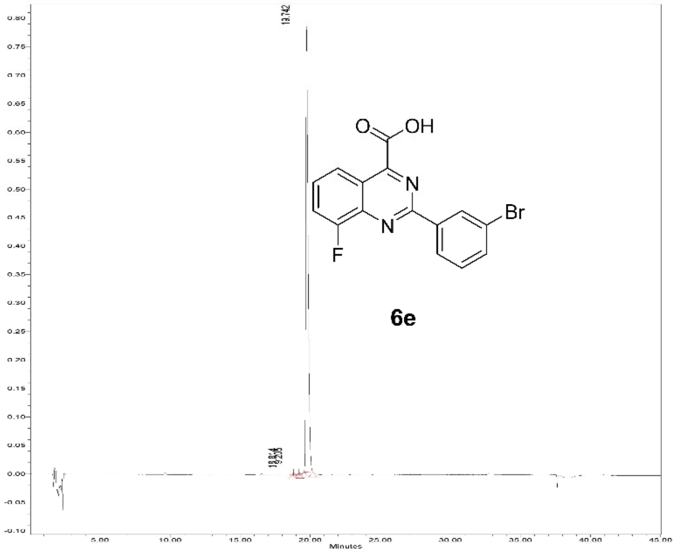

	Name	Retention Time (min)	Purity1 Angle	Purity1 Threshold	PDA Match1 Spect. Name	PDA Match1 Angle	PDA Match1 Threshold	PDA Match1 Lib. Name	Area (µV*sec)	% Area	Height (µV)	Int Type	Amount
1		22.062							9042	0.04	1651	bb	
2		23.523							20322738	99.96	2142776	bb	

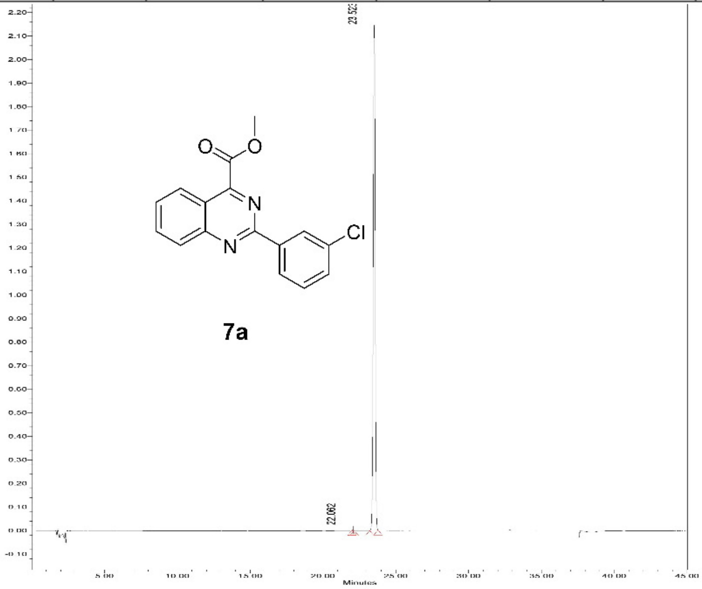

	Name	Retention Time (min)	Purity1 Angle	Purity1 Threshold	PDA Match1 Spect. Name	PDA Match1 Angle	PDA Match1 Threshold	PDA Match1 Lib. Name	Area (µV*sec)	% Area	Height (µV)	Int Type	Amount	Units
1		22.046							157671	2.48	16978	bb		
2		23.499							46452	0.73	6084	bb		
3		26.898							6157710	96.79	737125	bb		

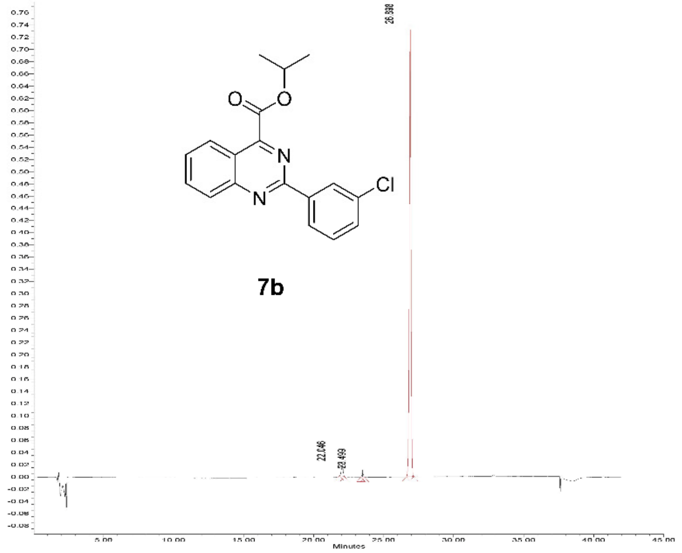

S1.3. Representative HRMS data.

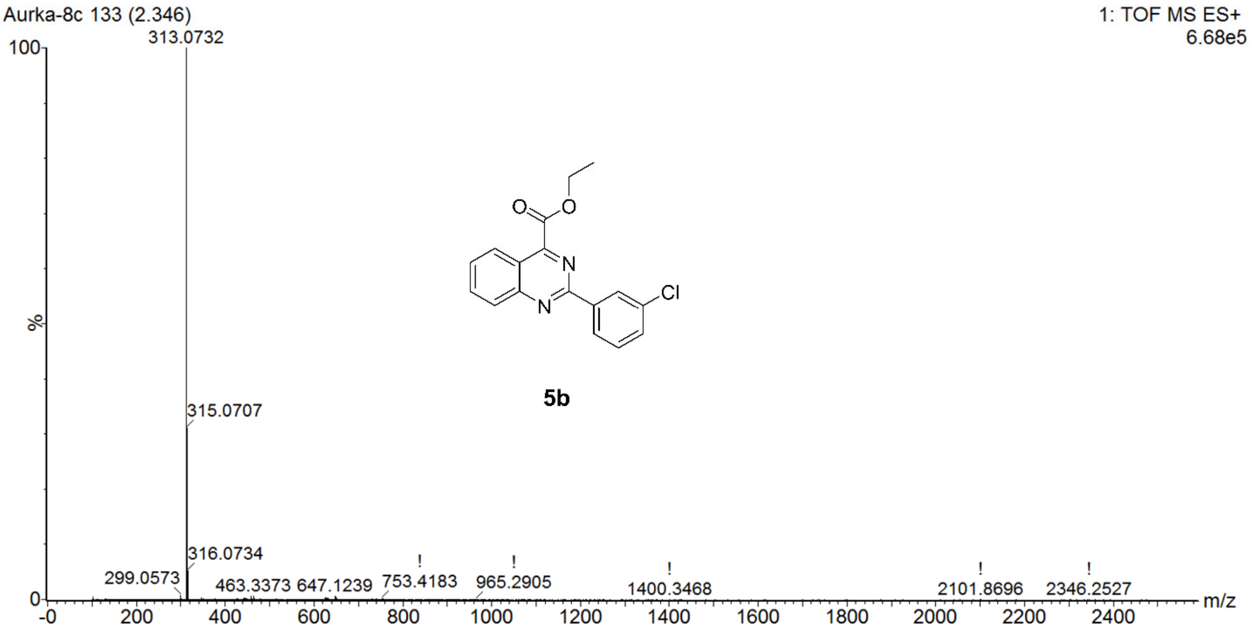

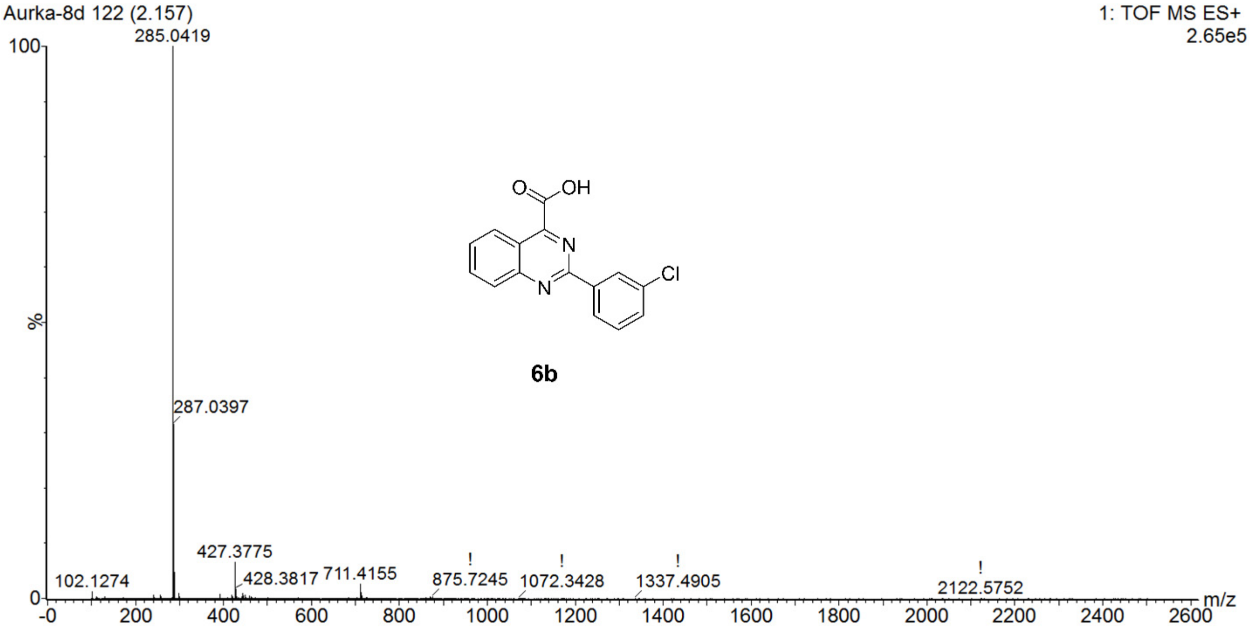

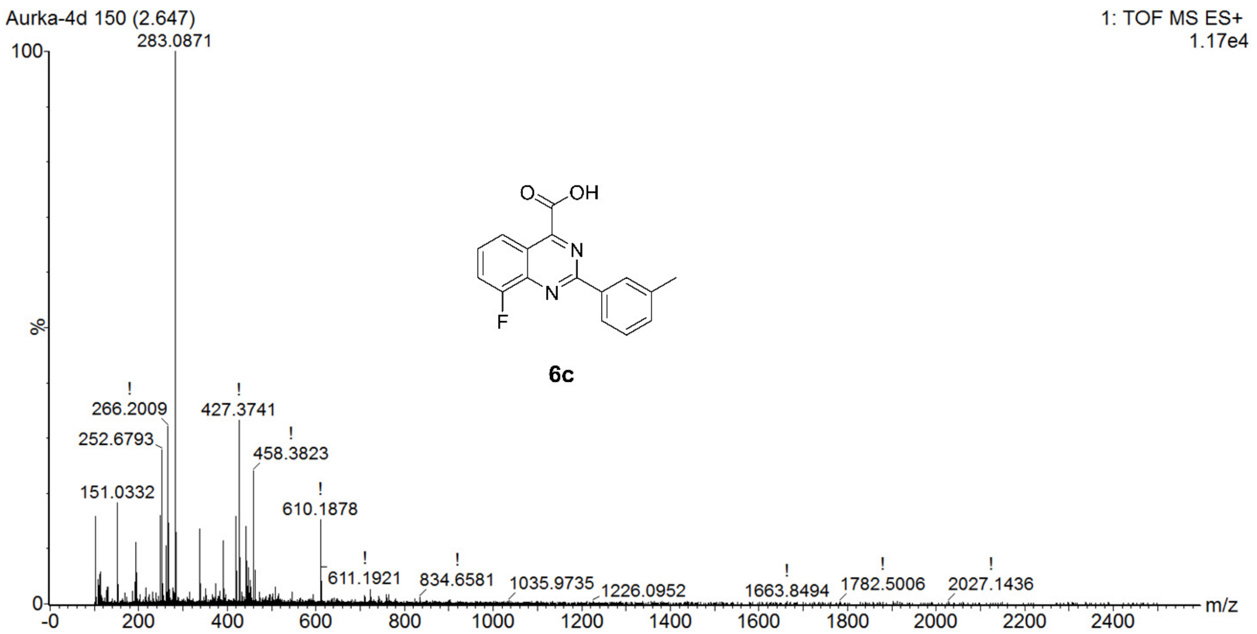
Aurka-4d 150 (2.647)
1: TOF MS ES+
1.17e4
283.0871
266.2009
252.6793
427.3741
458.3823
151.0332
610.1878
611.1921
834.6581
1035.9735
1226.0952
1663.8494
1782.5006
2027.1436
%
m/z
O
OH
N
N
F
6c

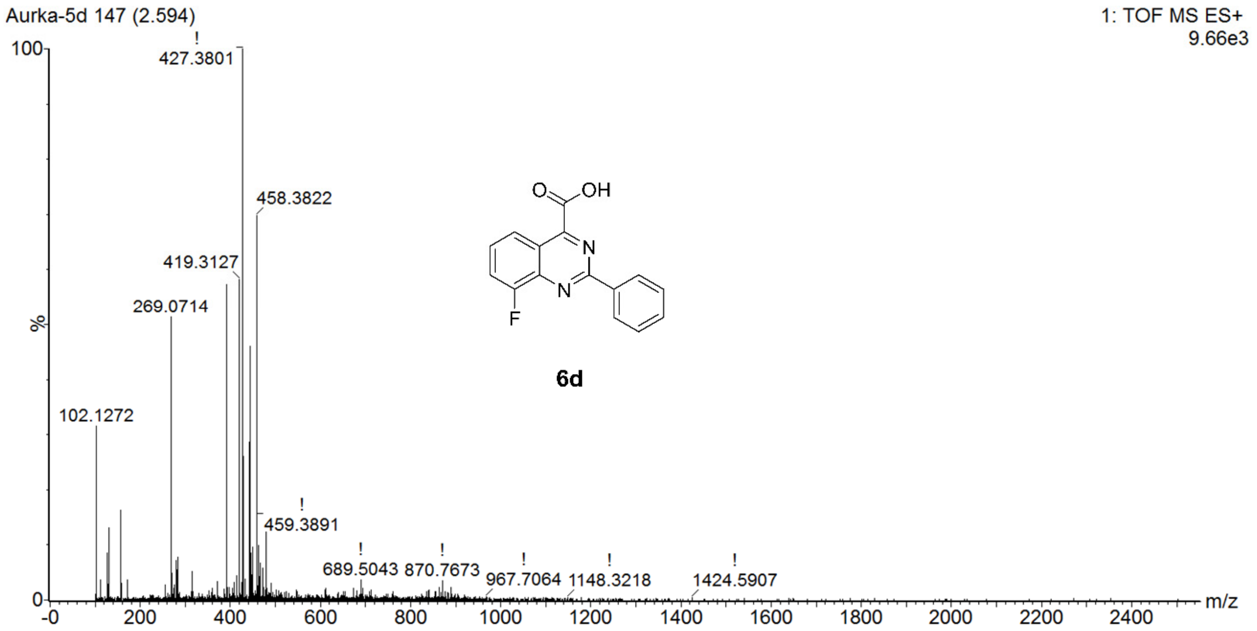
Aurka-5d 147 (2.594)
1: TOF MS ES+
9.66e3
427.3801
458.3822
419.3127
269.0714
102.1272
459.3891
689.5043
870.7673
967.7064
1148.3218
1424.5907
%
m/z
O
OH
N
N
F
6d

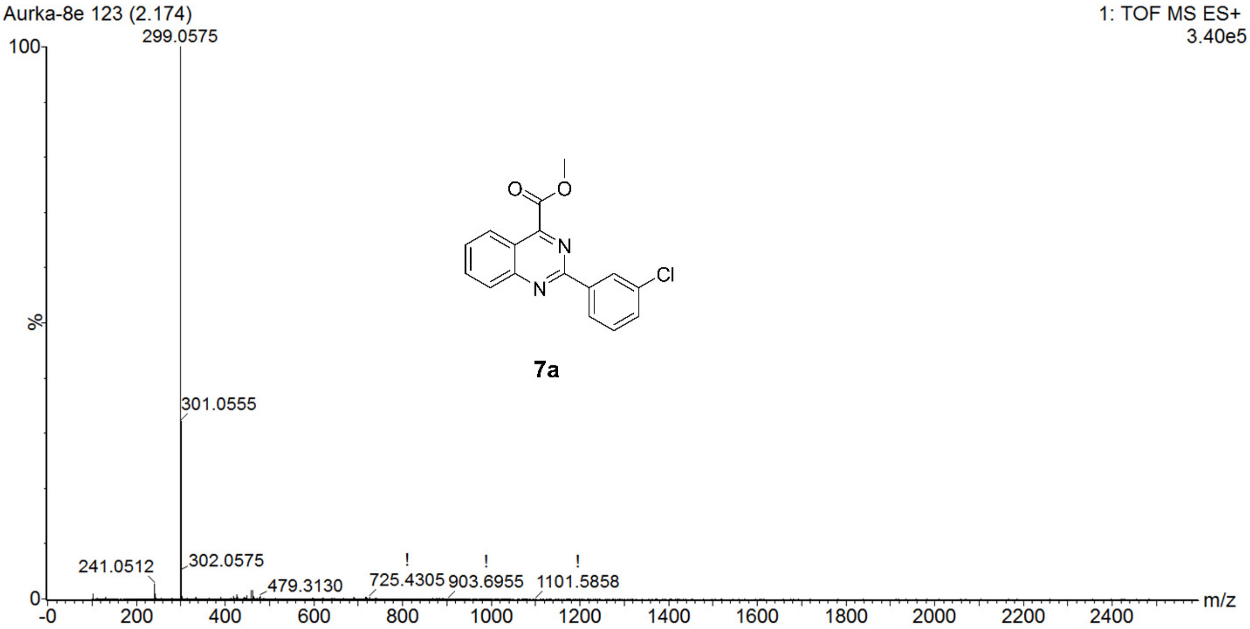
Aurka-8e 123 (2.174)
1: TOF MS ES+
3.40e5
299.0575
301.0555
241.0512
302.0575
479.3130
725.4305
903.6955
1101.5858
%
m/z
7a

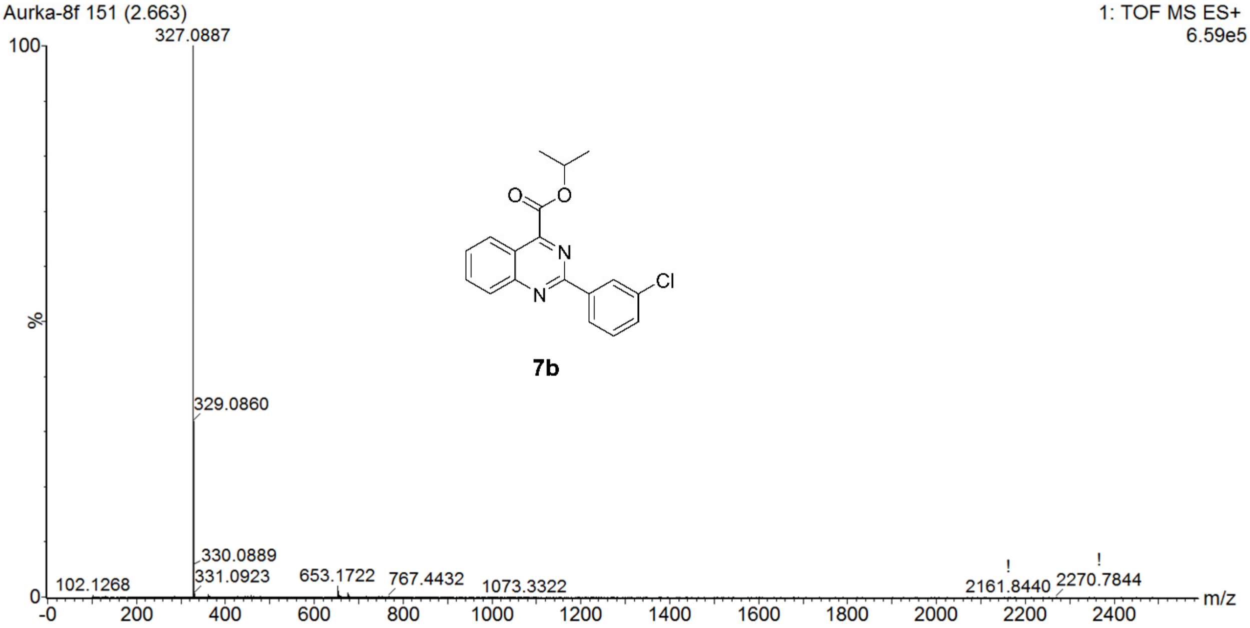
Aurka-8f 151 (2.663)
1: TOF MS ES+
6.59e5
327.0887
329.0860
330.0889
331.0923
102.1268
653.1722
767.4432
1073.3322
2161.8440
2270.7844
%
m/z
7b

S2. ***In vitro*** **screening**

Table S1. NCI results of compound **5b**.

**Developmental Therapeutics Program One Dose Mean Graph**	**NSC:** D-823457 / 1	**Conc:** 1.00E-5 Molar	**Test Date:** Mar 02, 2020
	**Experiment ID:** 2003OS23		**Report Date:** Mar 27, 2020

**Panel/Cell Line**	**Growth Percent**
Leukemia	
CCRF-CEM	98.71
HL-60(TB)	98.25
K-562	102.06
MOLT-4	94.14
RPMI-8226	100.15
SR	88.57
Non-Small Cell Lung Cancer	
A549/ATCC	94.97
EKVX	98.65
HOP-62	91.81
HOP-92	96.60
NCI-H226	105.86
NCI-H23	96.33
NCI-H322M	109.98
NCI-H460	101.55
NCI-H522	91.37
Colon Cancer	
COLO 205	111.49
HCC-2998	114.69
HCT-116	99.40
HCT-15	93.60
HT29	99.25
KM12	102.54
SW-620	100.66
CNS Cancer	
SF-268	98.18
SF-295	102.17
SF-539	102.07
SNB-19	91.46
SNB-75	64.77
U251	100.40
Melanoma	
LOX IMVI	99.45
MALME-3M	122.57
M14	109.47
MDA-MB-435	102.24
SK-MEL-2	108.22
SK-MEL-28	114.88
SK-MEL-5	101.32
UACC-257	108.15
UACC-62	84.33
Ovarian Cancer	
IGROV1	101.55
OVCAR-3	112.21
OVCAR-4	103.82
OVCAR-5	98.42
OVCAR-8	98.40
NCI/ADR-RES	104.37
SK-OV-3	102.24
Renal Cancer	
786-0	98.59
A498	75.74
ACHN	101.91
CAKI-1	94.65
RXF 393	97.15
SN12C	101.89
TK-10	81.84
UO-31	91.03
Prostate Cancer	
PC-3	102.57
DU-145	107.30
Breast Cancer	
MCF7	96.68
MDA-MB-231/ATCC	106.10
HS 578T	109.28
BT-549	85.98
T-47D	98.22
MDA-MB-468	105.83
Mean	99.60
Delta	34.83
Range	57.80

Mean Growth Percent - Growth Percent

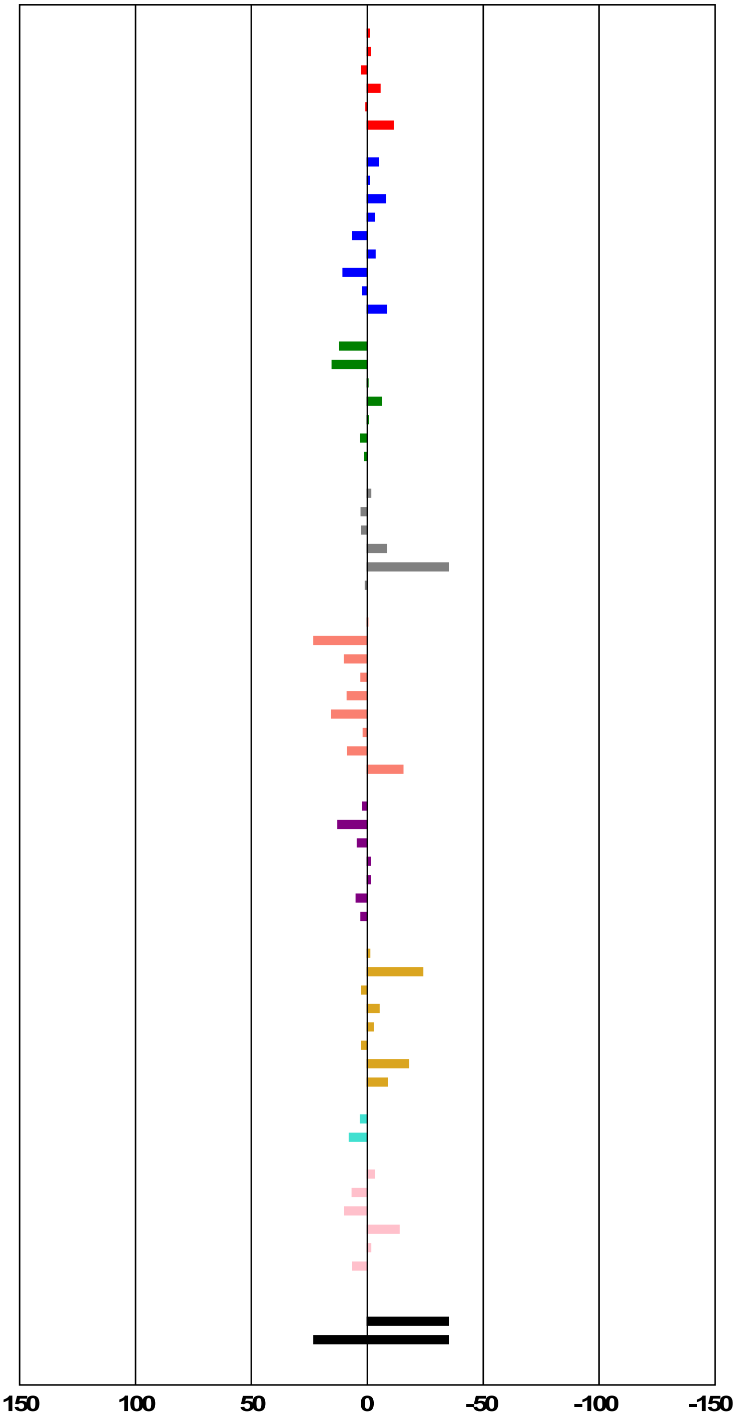

Table S2. NCI results of compound **6a**.

Developmental Therapeutics Program One Dose Mean Graph	**NSC:** D-823454 / 1	**Conc:** 1.00E-5 Molar	**Test Date:** Mar 02, 2020
	**Experiment ID:** 2003OS23		**Report Date:** Mar 27, 2020

Panel/Cell Line	Growth Percent
Leukemia	
CCRF-CEM	103.41
HL-60(TB)	97.39
K-562	97.94
MOLT-4	99.12
RPMI-8226	102.72
SR	85.62
Non-Small Cell Lung Cancer	
A549/ATCC	99.54
EKVX	102.12
HOP-62	96.76
HOP-92	102.20
NCI-H226	92.01
NCI-H23	91.36
NCI-H322M	109.61
NCI-H460	104.77
NCI-H522	101.36
Colon Cancer	
COLO 205	110.49
HCC-2998	99.35
HCT-116	102.14
HCT-15	105.62
HT29	108.78
KM12	104.82
SW-620	102.28
CNS Cancer	
SF-268	99.61
SF-295	102.48
SF-539	99.77
SNB-19	97.16
SNB-75	88.17
U251	105.36
Melanoma	
LOX IMVI	100.23
MALME-3M	102.52
M14	99.82
MDA-MB-435	101.61
SK-MEL-2	102.80
SK-MEL-28	104.98
SK-MEL-5	99.90
UACC-257	101.71
UACC-62	102.59
Ovarian Cancer	
IGROV1	100.96
OVCAR-3	106.44
OVCAR-4	105.22
OVCAR-5	99.36
OVCAR-8	97.97
NCI/ADR-RES	102.04
SK-OV-3	103.29
Renal Cancer	
786-0	99.76
A498	91.20
ACHN	101.47
CAKI-1	90.09
RXF 393	106.43
SN12C	97.44
TK-10	119.51
UO-31	87.43
Prostate Cancer	
PC-3	103.57
DU-145	105.12
Breast Cancer	
MCF7	92.28
MDA-MB-231/ATCC	108.22
HS 578T	107.49
BT-549	113.23
T-47D	107.42
MDA-MB-468	101.73
Mean	101.26
Delta	15.64
Range	33.89

Mean Growth Percent - Growth Percent

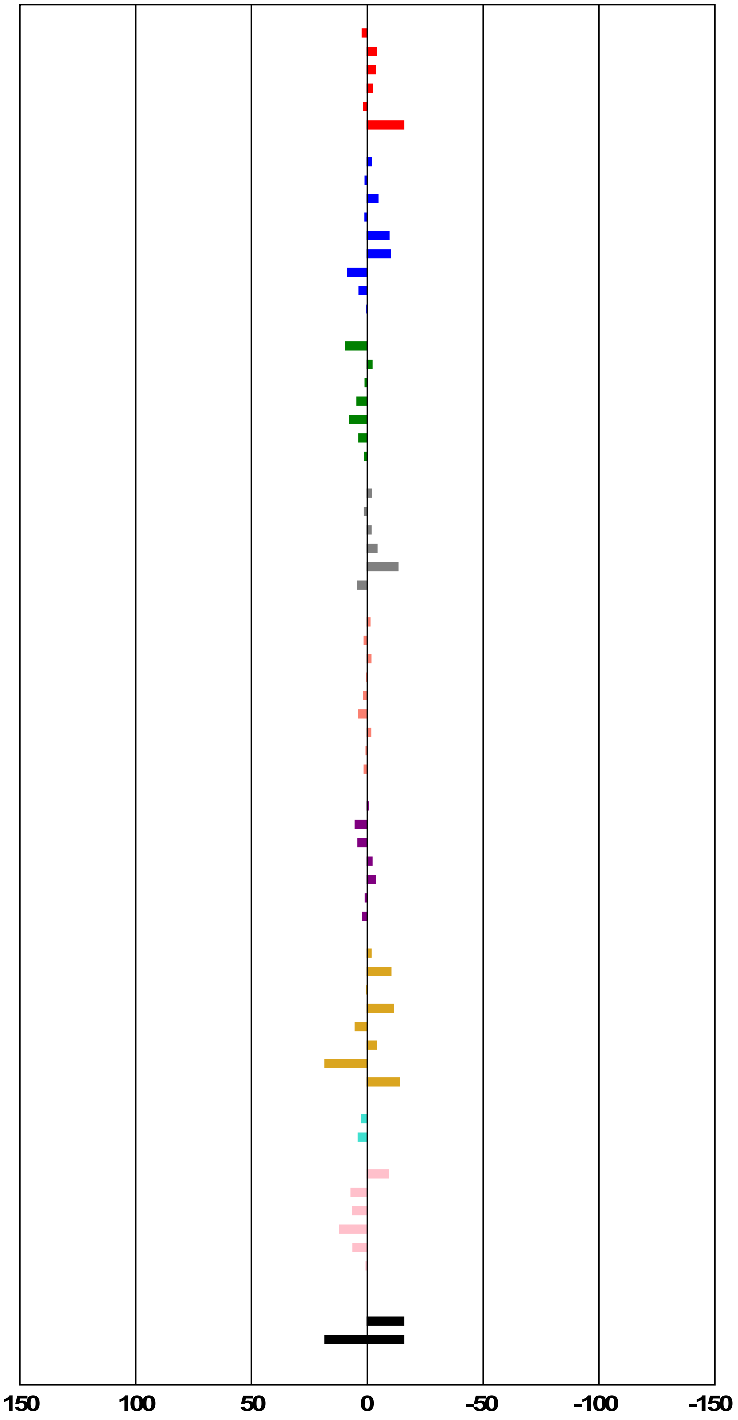

Table S3. NCI results of compound **6b**.

Developmental Therapeutics Program One Dose Mean Graph	**NSC:** D-823458 / 1	**Conc:** 1.00E-5 Molar	**Test Date:** Mar 02, 2020
	**Experiment ID:** 2003OS23		**Report Date:** Mar 27, 2020

Panel/Cell Line	Growth Percent
Leukemia	
CCRF-CEM	102.59
HL-60(TB)	93.74
K-562	104.62
MOLT-4	99.06
RPMI-8226	103.20
SR	103.19
Non-Small Cell Lung Cancer	
A549/ATCC	98.19
EKVX	98.55
HOP-62	93.17
HOP-92	87.64
NCI-H226	95.18
NCI-H23	93.82
NCI-H322M	105.03
NCI-H460	102.31
NCI-H522	92.66
Colon Cancer	
COLO 205	112.70
HCC-2998	106.17
HCT-116	100.86
HCT-15	97.87
HT29	110.32
KM12	100.23
SW-620	98.88
CNS Cancer	
SF-268	100.25
SF-295	100.35
SF-539	97.66
SNB-19	98.33
SNB-75	73.59
U251	99.25
Melanoma	
LOX IMVI	100.98
MALME-3M	104.95
M14	103.77
MDA-MB-435	101.25
SK-MEL-2	109.14
SK-MEL-28	107.98
SK-MEL-5	98.05
UACC-257	98.92
UACC-62	98.80
Ovarian Cancer	
IGROV1	101.76
OVCAR-3	108.68
OVCAR-4	98.39
OVCAR-5	95.74
OVCAR-8	96.83
NCI/ADR-RES	108.65
SK-OV-3	100.25
Renal Cancer	
786-0	101.35
A498	104.32
ACHN	98.55
CAKI-1	93.22
RXF 393	102.11
SN12C	99.19
TK-10	128.88
UO-31	78.64
Prostate Cancer	
PC-3	99.42
DU-145	109.97
Breast Cancer	
MCF7	87.41
MDA-MB-231/ATCC	96.54
HS 578T	107.07
BT-549	106.36
T-47D	103.52
MDA-MB-468	105.25
Mean	100.42
Delta	26.83
Range	55.29

Mean Growth Percent - Growth Percent

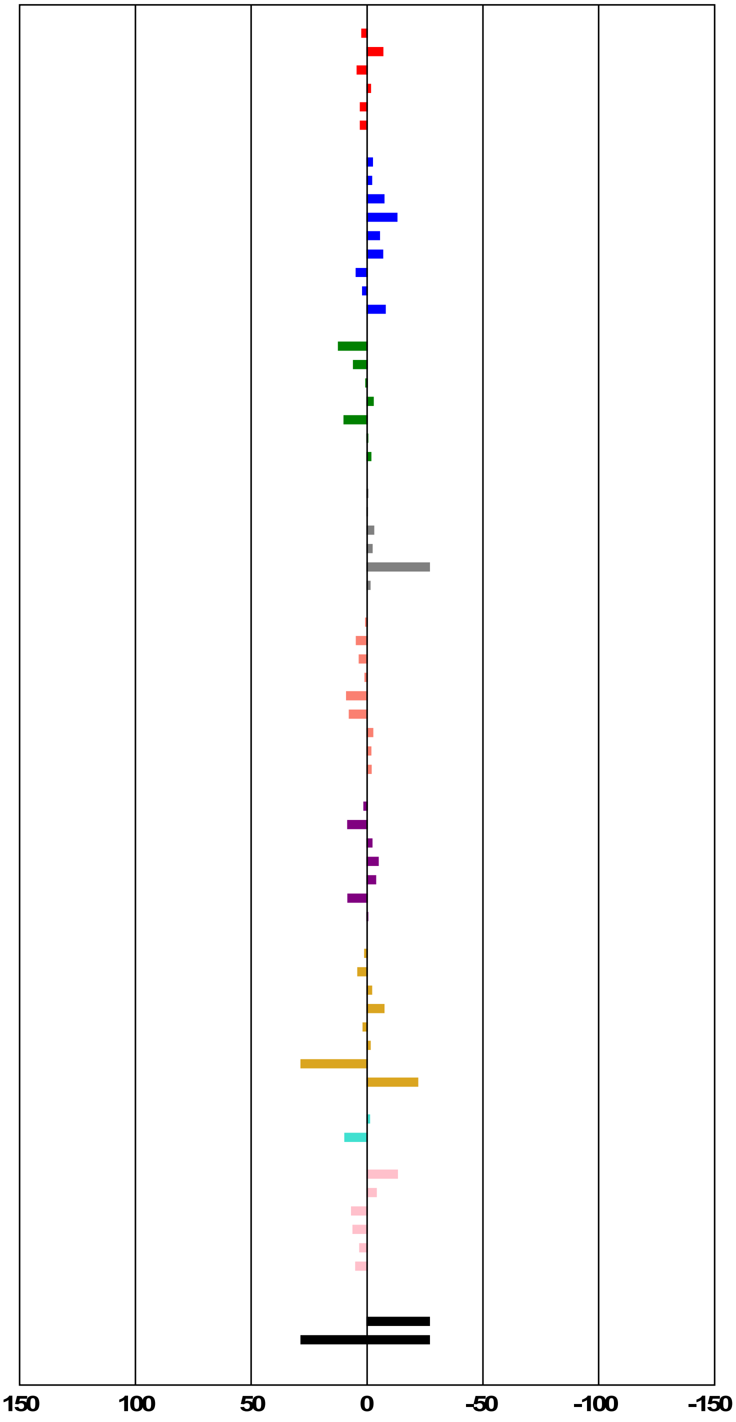

Table S4. NCI results of compound **6c**.

Developmental Therapeutics Program One Dose Mean Graph	**NSC:** D-823455 / 1	**Conc:** 1.00E-5 Molar	**Test Date:** Mar 02, 2020
	**Experiment ID:** 2003OS23		**Report Date:** Mar 27, 2020

Panel/Cell Line	Growth Percent
Leukemia	
CCRF-CEM	109.52
HL-60(TB)	100.43
K-562	99.54
MOLT-4	99.96
RPMI-8226	105.94
SR	90.77
Non-Small Cell Lung Cancer	
A549/ATCC	96.60
EKVX	104.27
HOP-62	95.25
HOP-92	109.58
NCI-H226	99.69
NCI-H23	96.37
NCI-H322M	106.44
NCI-H460	105.38
NCI-H522	96.52
Colon Cancer	
COLO 205	109.08
HCC-2998	103.16
HCT-116	98.50
HCT-15	99.91
HT29	106.12
KM12	102.90
SW-620	102.01
CNS Cancer	
SF-268	98.28
SF-295	101.08
SF-539	102.59
SNB-19	97.50
SNB-75	79.79
U251	104.72
Melanoma	
LOX IMVI	96.81
MALME-3M	105.74
M14	102.57
MDA-MB-435	99.63
SK-MEL-2	104.72
SK-MEL-28	107.19
SK-MEL-5	100.75
UACC-257	96.22
UACC-62	106.79
Ovarian Cancer	
IGROV1	106.82
OVCAR-3	112.72
OVCAR-4	90.44
OVCAR-5	101.55
OVCAR-8	98.54
NCI/ADR-RES	104.94
SK-OV-3	101.53
Renal Cancer	
786-0	99.80
A498	97.16
ACHN	105.94
CAKI-1	97.67
RXF 393	105.05
SN12C	107.04
TK-10	98.81
UO-31	89.61
Prostate Cancer	
PC-3	112.26
DU-145	110.28
Breast Cancer	
MCF7	94.33
MDA-MB-231/ATCC	106.53
HS 578T	105.70
BT-549	98.91
T-47D	102.94
MDA-MB-468	95.85
Mean	101.45
Delta	21.66
Range	32.93

Mean Growth Percent - Growth Percent

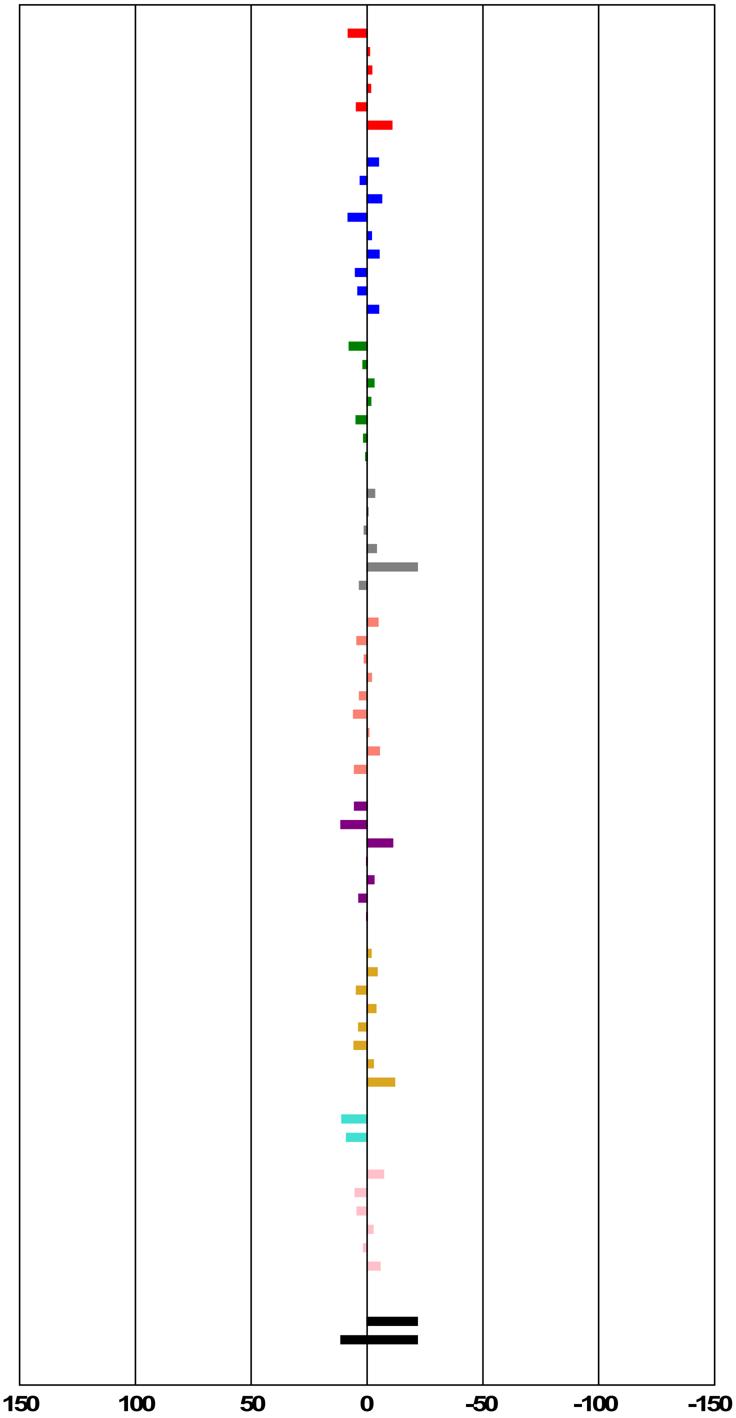

Table S5. NCI results of compound **6d**.

Developmental Therapeutics Program One Dose Mean Graph	NSC: D-823456 / 1	Conc: 1.00E-5 Molar	Test Date: Mar 02, 2020
	Experiment ID: 2003OS23		Report Date: Mar 27, 2020

Panel/Cell Line	Growth Percent
**Leukemia**	
CCRF-CEM	101.08
HL-60(TB)	95.82
K-562	97.12
MOLT-4	100.92
RPMI-8226	102.34
SR	94.90
**Non-Small Cell Lung Cancer**	
A549/ATCC	97.31
EKVX	91.20
HOP-62	87.56
HOP-92	103.21
NCI-H226	94.79
NCI-H23	92.49
NCI-H322M	107.72
NCI-H460	104.48
NCI-H522	91.01
**Colon Cancer**	
COLO 205	105.80
HCC-2998	102.53
HCT-116	105.19
HCT-15	98.34
HT29	105.15
KM12	103.39
SW-620	99.78
**CNS Cancer**	
SF-268	100.05
SF-295	101.26
SF-539	98.97
SNB-19	96.00
SNB-75	82.18
U251	97.24
**Melanoma**	
LOX IMVI	96.69
MALME-3M	108.06
M14	103.84
MDA-MB-435	101.51
SK-MEL-2	110.11
SK-MEL-28	103.08
SK-MEL-5	101.05
UACC-257	95.74
UACC-62	100.74
**Ovarian Cancer**	
IGROV1	88.49
OVCAR-3	115.79
OVCAR-4	94.94
OVCAR-5	103.40
OVCAR-8	97.84
NCI/ADR-RES	104.53
SK-OV-3	96.71
**Renal Cancer**	
786-0	103.46
A498	101.92
ACHN	102.98
CAKI-1	93.16
RXF 393	105.65
SN12C	97.15
TK-10	149.35
UO-31	85.80
**Prostate Cancer**	
PC-3	99.43
DU-145	110.91
**Breast Cancer**	
MCF7	82.33
MDA-MB-231/ATCC	96.92
HS 578T	103.93
BT-549	105.10
T-47D	101.76
MDA-MB-468	110.82
Mean	100.52
Delta	18.34
Range	67.17

Mean Growth Percent - Growth Percent

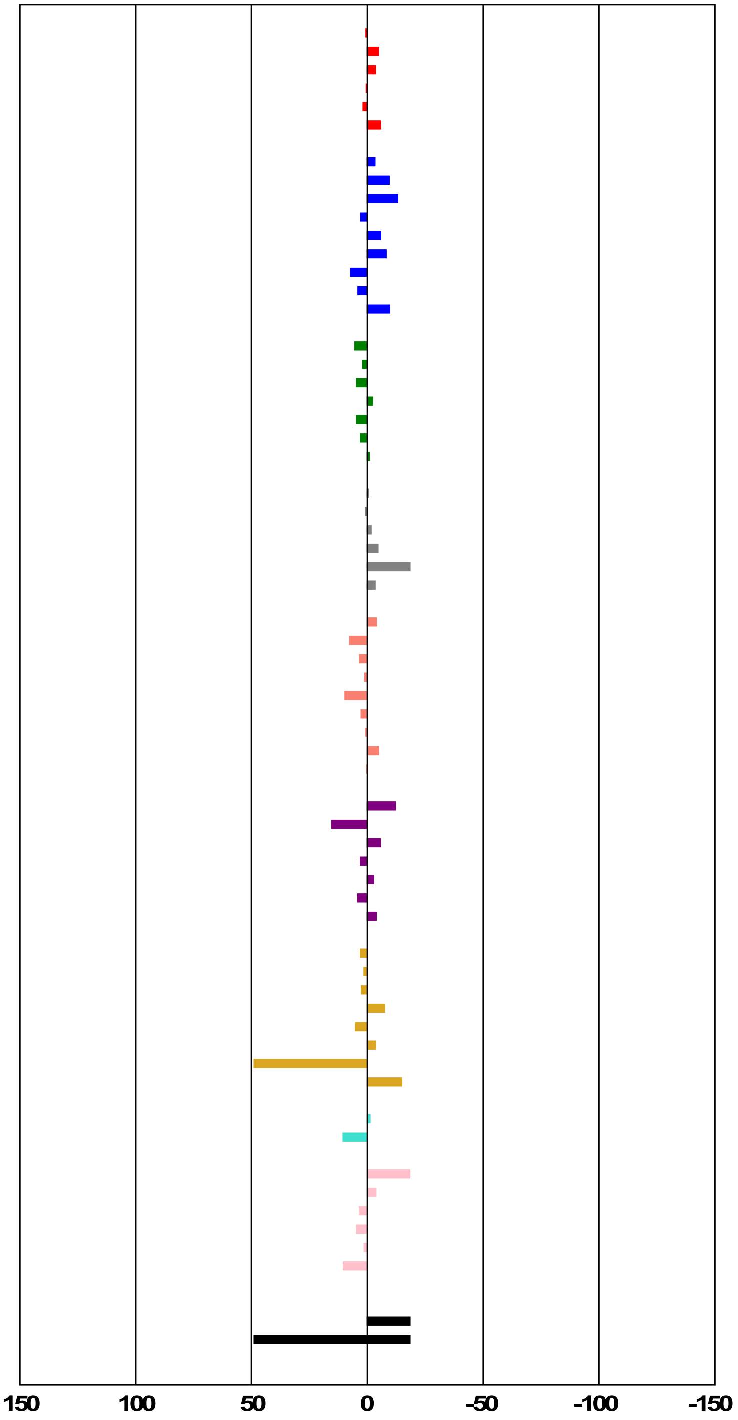

Table S6. NCI results of compound **6e**.

Developmental Therapeutics Program One Dose Mean Graph	**NSC:** D-823453 / 1	**Conc:** 1.00E-5 Molar	**Test Date:** Mar 02, 2020
	**Experiment ID:** 2003OS23		**Report Date:** Mar 27, 2020

Panel/Cell Line	Growth Percent
Leukemia	
CCRF-CEM	101.68
HL-60(TB)	103.81
K-562	107.74
MOLT-4	106.44
RPMI-8226	98.60
SR	104.82
Non-Small Cell Lung Cancer	
A549/ATCC	105.61
EKVX	99.85
HOP-62	89.71
HOP-92	90.62
NCI-H226	95.41
NCI-H23	96.38
NCI-H322M	102.39
NCI-H460	103.14
NCI-H522	91.58
Colon Cancer	
COLO 205	113.16
HCC-2998	101.36
HCT-116	99.54
HCT-15	104.18
HT29	98.25
KM12	101.02
SW-620	99.91
CNS Cancer	
SF-268	98.31
SF-295	103.28
SF-539	98.35
SNB-19	93.55
SNB-75	66.99
U251	100.62
Melanoma	
LOX IMVI	97.48
MALME-3M	103.45
M14	99.76
MDA-MB-435	105.45
SK-MEL-2	112.47
SK-MEL-28	104.67
SK-MEL-5	101.05
UACC-257	106.87
UACC-62	100.89
Ovarian Cancer	
IGROV1	99.30
OVCAR-3	120.73
OVCAR-4	104.25
OVCAR-5	98.08
OVCAR-8	103.62
NCI/ADR-RES	102.61
SK-OV-3	95.51
Renal Cancer	
786-0	98.13
A498	97.81
ACHN	101.76
CAKI-1	93.94
RXF 393	102.45
SN12C	94.87
TK-10	106.16
UO-31	88.96
Prostate Cancer	
PC-3	102.98
DU-145	111.98
Breast Cancer	
MCF7	89.88
MDA-MB-231/ATCC	105.28
HS 578T	110.78
BT-549	109.68
T-47D	104.92
MDA-MB-468	98.86
Mean	100.85
Delta	33.86
Range	53.74

Mean Growth Percent - Growth Percent

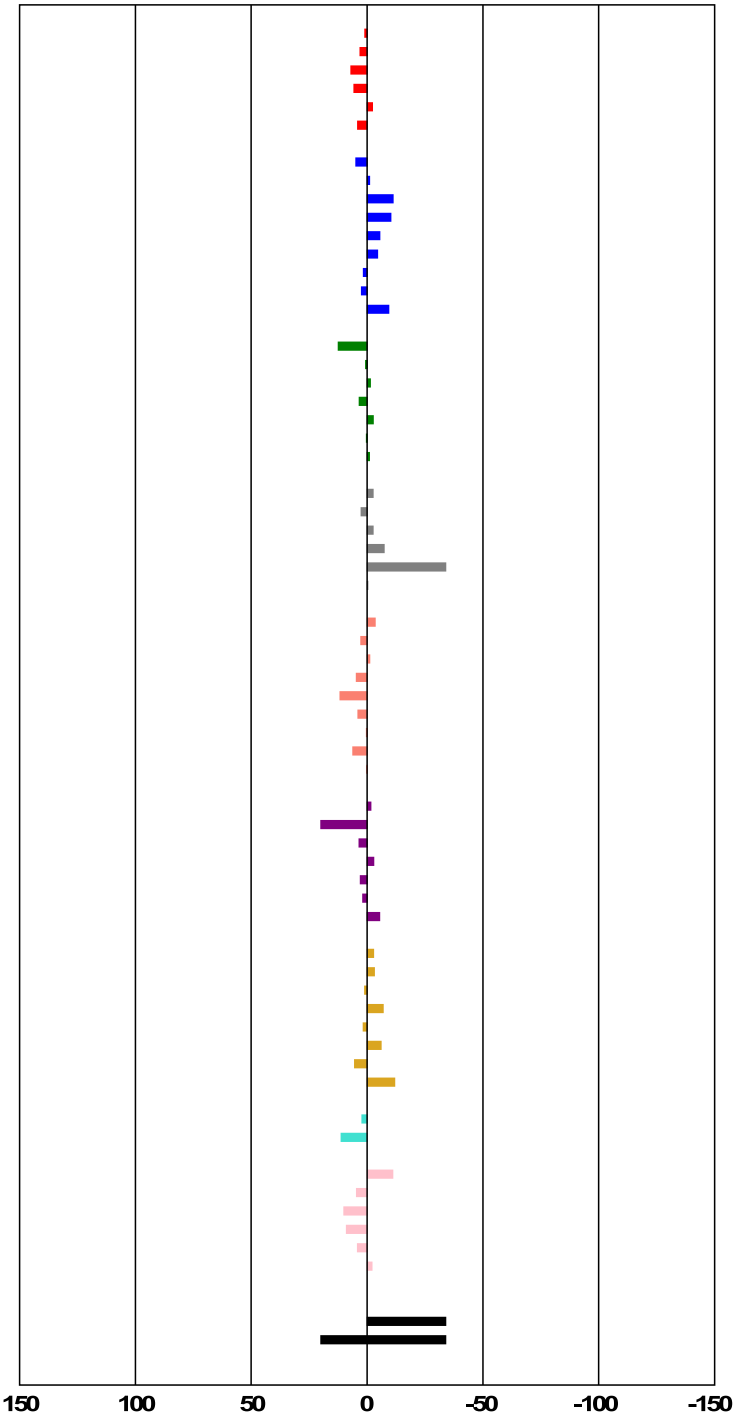

Table S7. NCI results of compound **7a**.

Developmental Therapeutics Program One Dose Mean Graph	NSC: D-823459 / 1	Conc: 1.00E-5 Molar	Test Date: Mar 02, 2020
	Experiment ID: 2003OS23		Report Date: Mar 27, 2020

Panel/Cell Line	Growth Percent
Leukemia	
CCRF-CEM	98.98
HL-60(TB)	98.12
K-562	93.04
MOLT-4	97.65
RPMI-8226	102.63
SR	92.35
Non-Small Cell Lung Cancer	
A549/ATCC	95.50
EKVX	86.24
HOP-62	95.47
HOP-92	91.68
NCI-H226	91.16
NCI-H23	85.04
NCI-H322M	101.87
NCI-H460	104.11
NCI-H522	93.37
Colon Cancer	
COLO 205	112.89
HCC-2998	107.88
HCT-116	101.87
HCT-15	100.28
HT29	105.81
KM12	98.41
SW-620	101.04
CNS Cancer	
SF-268	96.60
SF-295	102.68
SF-539	100.51
SNB-19	92.09
SNB-75	80.12
U251	100.89
Melanoma	
LOX IMVI	96.90
MALME-3M	108.90
M14	108.38
MDA-MB-435	101.52
SK-MEL-2	111.61
SK-MEL-28	103.46
SK-MEL-5	100.65
UACC-257	102.34
UACC-62	91.73
Ovarian Cancer	
IGROV1	92.07
OVCAR-3	110.29
OVCAR-4	87.74
OVCAR-5	103.74
OVCAR-8	99.14
NCI/ADR-RES	102.57
SK-OV-3	106.87
Renal Cancer	
786-0	101.54
A498	87.08
ACHN	101.21
CAKI-1	95.36
RXF 393	106.18
SN12C	97.20
TK-10	160.35
UO-31	84.57
Prostate Cancer	
PC-3	103.50
DU-145	108.33
Breast Cancer	
MCF7	67.18
MDA-MB-231/ATCC	89.84
HS 578T	113.41
BT-549	99.79
T-47D	94.15
MDA-MB-468	104.50
Mean	99.51
Delta	32.33
Range	93.17

Mean Growth Percent - Growth Percent

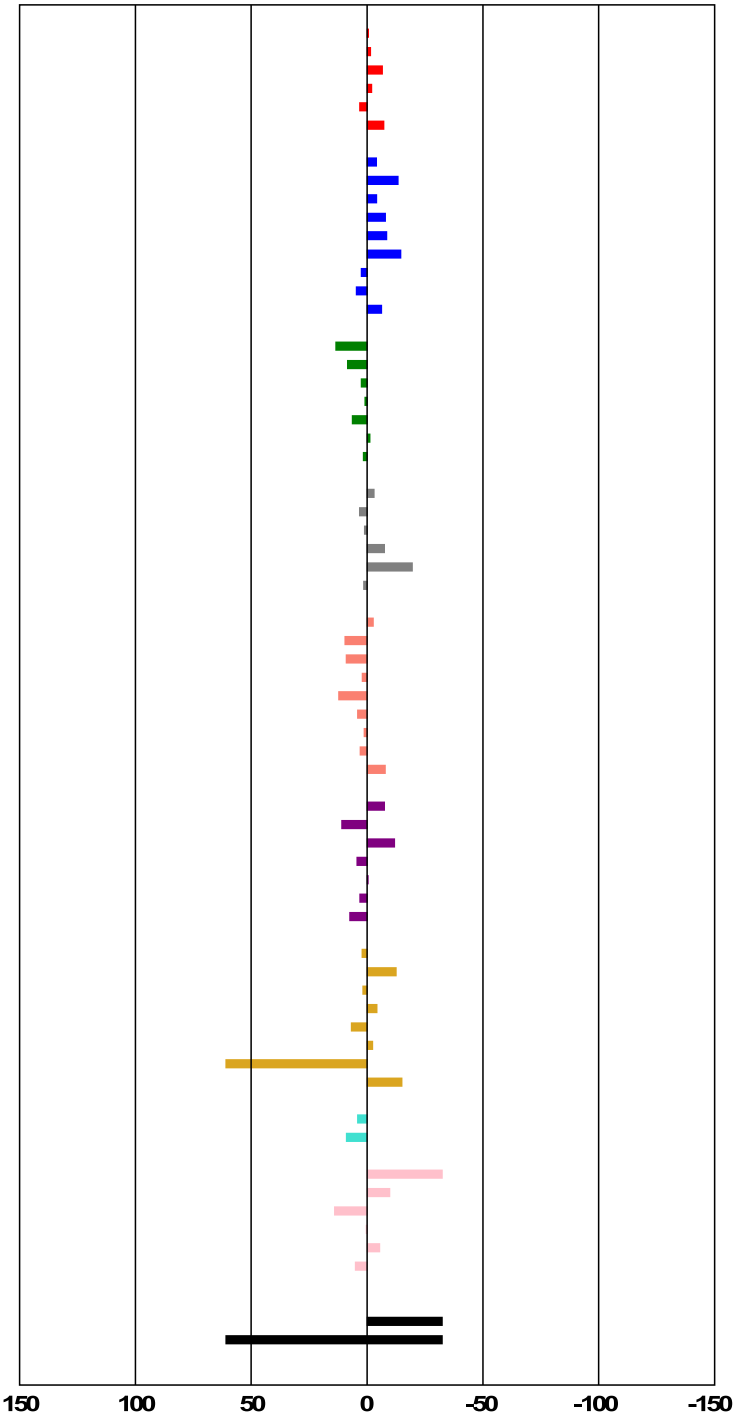

Table S8. NCI results of compound **7b**.

Developmental Therapeutics Program One Dose Mean Graph	NSC: D-823460 / 1	Conc: 1.00E-5 Molar	Test Date: Mar 02, 2020
	Experiment ID: 2003OS23		Report Date: Mar 27, 2020

Panel/Cell Line	Growth Percent
Leukemia	
CCRF-CEM	101.82
HL-60(TB)	101.46
K-562	92.88
MOLT-4	96.11
RPMI-8226	100.75
SR	97.85
Non-Small Cell Lung Cancer	
A549/ATCC	100.03
EKVX	94.19
HOP-62	94.69
HOP-92	104.54
NCI-H226	102.42
NCI-H23	89.87
NCI-H322M	111.03
NCI-H460	104.89
NCI-H522	91.72
Colon Cancer	
COLO 205	107.44
HCC-2998	118.61
HCT-116	105.66
HCT-15	99.03
HT29	101.41
KM12	100.17
SW-620	104.38
CNS Cancer	
SF-268	99.73
SF-295	103.87
SF-539	104.37
SNB-19	100.22
SNB-75	75.14
U251	101.47
Melanoma	
LOX IMVI	100.17
MALME-3M	136.07
M14	118.45
MDA-MB-435	108.18
SK-MEL-2	104.73
SK-MEL-28	103.31
SK-MEL-5	101.87
UACC-257	106.55
UACC-62	87.90
Ovarian Cancer	
IGROV1	98.48
OVCAR-3	111.14
OVCAR-4	102.20
OVCAR-5	103.15
OVCAR-8	102.12
NCI/ADR-RES	105.64
SK-OV-3	103.97
Renal Cancer	
786-0	101.61
A498	83.49
ACHN	100.81
CAKI-1	97.29
RXF 393	110.50
SN12C	100.63
TK-10	143.46
UO-31	92.22
Prostate Cancer	
PC-3	111.37
DU-145	104.64
Breast Cancer	
MCF7	81.39
MDA-MB-231/ATCC	100.99
HS 578T	108.92
BT-549	116.67
T-47D	93.84
MDA-MB-468	108.38
Mean	102.60
Delta	27.46
Range	68.32

Mean Growth Percent - Growth Percent

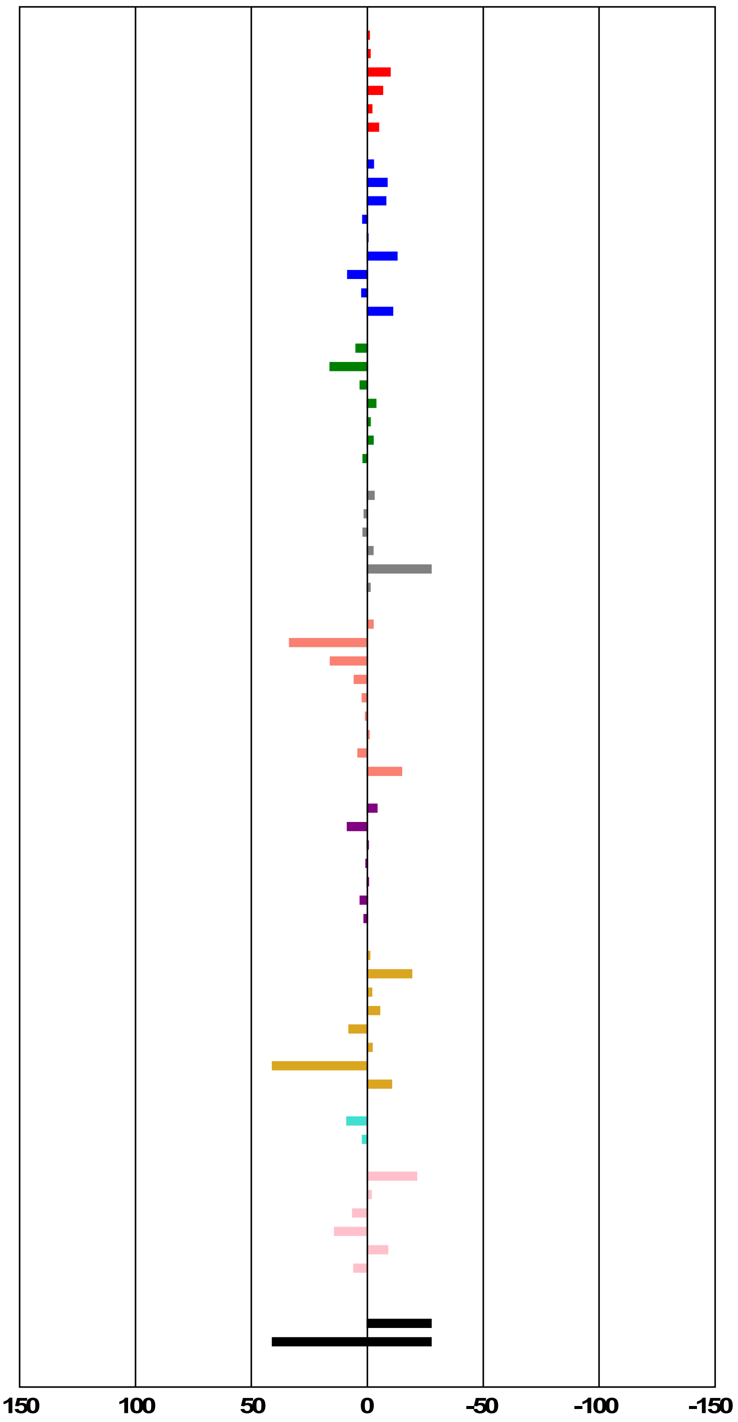

Supplement: Supplementary file 1 [file life-12-00876-s001.zip › life-1721180-supplementary.pdf]
